# Real‐world treatment patterns and clinical outcomes after introduction of immune checkpoint inhibitors: Results from a retrospective chart review of patients with advanced/metastatic non‐small cell lung cancer in the EU5

**DOI:** 10.1111/1759-7714.15069

**Published:** 2023-08-17

**Authors:** Alexander Slowley, Kelesitse Phiri, Jasjit K. Multani, Vicky Casey, Sheila Mpima, Marie Yasuda, Chi‐Chang Chen, Fil Manuguid, Jessica Chao, Amine Aziez, Kelly F. Bell, Alexander Stojadinovic

**Affiliations:** ^1^ GSK London UK; ^2^ GSK Collegeville Pennsylvania USA; ^3^ IQVIA Falls Church Virginia USA; ^4^ IQVIA London UK; ^5^ IQVIA Wayne Pennsylvania USA; ^6^ GSK Upper Providence Pennsylvania USA; ^7^ GSK Zug Switzerland

**Keywords:** immune checkpoint inhibition, metastases, neoplasm, non‐small cell lung cancer, retrospective studies

## Abstract

**Background:**

Real‐world evidence is increasingly used to guide treatment and regulatory decisions for non‐small cell lung cancer (NSCLC). Real‐world treatment patterns and clinical outcomes among patients with advanced/metastatic NSCLC in France, Germany, Italy, Spain, and the UK (EU5) were assessed.

**Methods:**

This retrospective physician‐completed patient chart review assessed treatment patterns (regimen, duration of treatment [DOT], time to discontinuation), and clinical outcomes (duration of response [DOR], progression‐free survival [PFS], and overall survival [OS]) of patients with stage IIIB/C or IV NSCLC who received pembrolizumab‐based first‐line induction chemotherapy.

**Results:**

Overall, 322 patients were included; at first‐line maintenance (1LM), 92% had stage IV NSCLC, 68% had nonsquamous histology, and 89% had no central nervous system (CNS)/brain metastasis. The two most common 1LM regimens were pembrolizumab monotherapy (76% overall) and pembrolizumab + pemetrexed (21% overall). Docetaxel monotherapy was the most common second‐line regimen in all countries except Germany (54% overall). For 1LM therapy, the overall median DOT and DOR were 5 and 10 months, respectively; PFS was 7 months and OS was 8 months. Germany had a longer duration of each outcome except for DOR which was longer in Spain. Clinical outcomes were generally poorer for patients with squamous histology and CNS/brain metastases.

**Conclusions:**

This study demonstrated differences in treatment patterns and clinical outcomes in NSCLC across the EU5 and patient subgroups. Improved survival was generally associated with response to first‐line therapy, nonsquamous histology, and CNS/brain metastases absence. These real‐world data provide valuable insights which may aid treatment decision‐making and clinical trial design.

## Graphical abstract

This retrospective study provides real‐world treatment patterns and clinical outcomes among patients with advanced/metastatic non‐small cell lung cancer (NSCLC) in France, Germany, Italy, Spain, and the UK (EU5). The most common first line maintenance regimen was pembrolizumab monotherapy, pembrolizumab + pemetrexed was the second most‐common. This study also highlights differences in treatment patterns and clinical outcomes across the EU5, providing insight into real‐world clinical practice which may aid future treatment decision‐making and clinical trial design.

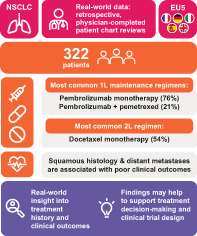

## INTRODUCTION

Non‐small cell lung cancer (NSCLC) treatment has advanced greatly over the past two decades due to treatments targeting driver alterations in genes including anaplastic lymphoma kinase (ALK), B‐Raf (BRAF), and epidermal growth factor receptor (EGFR).^1^ However, treatment of patients with tumors that do not harbor such alterations remains a challenge.^1^ NSCLC treatment predominantly involves immune checkpoint inhibitors (ICIs), specifically programmed death‐1/programmed death ligand‐1 (PD‐1/PD‐L1) inhibitors^1^ along with chemotherapy. Current guidelines from the European Society for Medical Oncology (ESMO) recommend immunotherapy with anti‐PD‐1 antibody pembrolizumab as a first‐line (1L) therapy, with or without platinum‐based chemotherapy (biomarker status‐dependent).^2^ Following 1L induction therapy, patients are recommended to undergo 1L maintenance (1LM) therapy, which involves either continuation of 1L induction agent(s) (continuation maintenance) or initiation of a different agent following completion of induction chemotherapy (switch maintenance).^2^

The objective of this study was to analyze real‐world data using retrospective patient chart review to understand demographics, clinical characteristics, treatment patterns, and clinical outcomes for patients with advanced/metastatic NSCLC receiving 1LM therapy in France, Germany, Italy, Spain, and the UK (termed EU5 henceforth).

## METHODS

### Study design

This descriptive retrospective patient chart review – based on a standardized, online physician‐completed case report form (CRF) – captured anonymous patient data and treatment history since diagnosis of NSCLC. Questions on cross‐sectional CRFs were completed by a panel of physicians for each country from July–August, 2021. Index date was the start of 1LM therapy. Treatment history for both living and deceased patients was captured.

### Data source/data collection

Physicians were recruited from a proprietary internal IQVIA database of healthcare providers. Screening was based on experience, workload, and management of patients with NSCLC. Physicians included clinical/medical and surgical oncologists, drug‐prescribing radiation oncologists, pulmonologists, and thoracic surgeons, who were required to have personally treated a patient with advanced/metastatic NSCLC on 1LM therapy ≤12‐months prior to or at time of data extraction. A maximum of two physicians from the same center and one physician from the same hospital, per country, were permitted to participate in CRF collection, to avoid cluster effects and patient duplication.

### Study population

Eligible patients were ≥18 years old at index; had histologically/cytologically confirmed diagnosis of squamous, nonsquamous, or mixed‐histology NSCLC (defined by the American Joint Committee on Cancer eighth edition Staging Manual as advanced [stage IIIB, not amenable to definitive chemoradiotherapy or stage IIIC] or metastatic [stage IV]) between January 1, 2018 and December 31, 2020; completed 4–6 cycles of platinum‐based 1L induction chemotherapy with pembrolizumab (standard of care per National Comprehensive Cancer Network and ESMO Clinical Practice Guidelines^2^, ^3^), achieved stable disease (SD), partial response (PR), or complete response (CR) per physician's assessment, prior to initiation of pembrolizumab‐based 1LM therapy and had an Eastern Cooperative Oncology Group performance stage (ECOG PS) of 0–1 at index or at closest assessment prior to index (patients with missing ECOG PS or date of birth were excluded). Additional exclusion criteria were the presence of targetable driver mutations in ALK, EGFR, BRAF, ROS proto‐oncogene 1, neurotrophic tyrosine receptor kinase, mesenchymal epithelial transition factor receptor, or Erb‐B2 receptor tyrosine kinase 2, regardless of PD‐L1 status; receipt of chemoradiotherapy ≤6‐months prior to initiation of 1LM; leptomeningeal disease, carcinomatous meningitis, symptomatic brain metastases or radiological signs of central nervous system (CNS) hemorrhage at index; known history or current diagnosis of myelodysplastic syndrome or acute myeloid leukemia; receipt of systemic cytotoxic chemotherapy, biological therapy, or hormonal therapy for cancer prior to NSCLC diagnosis; or pregnancy during or after advanced/metastatic NSCLC diagnosis.

### Study outcomes

Study outcomes included baseline demographic and clinical characteristics of patients with advanced/metastatic NSCLC receiving 1LM therapy, treatment patterns including the drug regimens used in each line of therapy (LOT; 1L induction, 1LM, second‐line [2L], third‐line [3L]), duration of treatment (DOT), proportion of patients with discontinuation, time to discontinuation (TTD), reasons for discontinuation, and real‐world time to next treatment (rwTTNT). Clinical outcomes included progression‐free survival (PFS), time to CNS/brain progression and overall survival (OS) from start of 1LM as well as objective response rate (ORR; the proportion of patients with a PR or CR), and duration of response (DOR) after completion of 1L induction. Exploratory clinical outcomes were time to CNS/brain progression from start of 1L induction therapy and time to death from start of 1LM therapy. Timelines for the assessment of outcomes are depicted in Figure S1.

### Data handling

Data collection complied with all applicable laws and regulations, including GDPR (Regulation [EU] 2016/679 of the European Parliament and the Council on the Protection of individuals with regard to the processing of personal data and on the free movement of such data), as well as applicable codes and guidelines.

### Data analysis

All analyses were performed using RStudio version 3.6.2 statistical software. Descriptive statistics were used to report patient demographics and clinical characteristics. For subgroup analyses, patients were stratified into subgroups based on: country, histology (squamous vs. nonsquamous vs. mixed histology), disease stage (advanced vs. metastatic), PD‐L1 status (tumor proportion score [TPS]‐negative [<1%] vs. ≥1% and/or 1–49% vs. ≥50%), best response to induction chemotherapy (CR/PR vs. SD), pemetrexed use in 1LM therapy (yes vs. no; nonsquamous only), and asymptomatic CNS/brain metastases at the start of 1LM therapy (yes vs. no). Where applicable, N (%) of patients with missing data were included in the “unknown/missing” response option. No imputation methods were used to handle missing data. Continuous variables were evaluated to determine normality and to identify outliers. Time to event analyses (DOT, TTD, rwTTNT, time to CNS/brain progression) were reported using Kaplan–Meier survival methods and analyzed by log‐rank test. Censored events included death and end of follow‐up. Patients were censored at the earliest of these events if they occurred before observation of the outcome of interest.

## RESULTS

### Patient characteristics

A total of 322 patients with advanced/metastatic NSCLC were included: (France, *n* = 61; Germany, *n* = 60; Italy, *n* = 73; Spain, *n* = 67; and UK, *n* = 61) (Table 1). Most patients, (97%), were aged >45 years at index. Baseline characteristics were similar across countries, with a few exceptions: ≥49% of patients were aged 66–75 years in France, Germany, and the UK, whereas only 37% and 40% of patients were in this age range in Italy and Spain. Germany had the highest proportion (17%) of patients aged ≥76, while Spain had the highest proportion (7%) of patients aged ≤45.

**TABLE 1:** Patient characteristics.

Characteristic	All (*N* = 322)	France (*N* = 61)	Germany (*N* = 60)	Italy (*N* = 73)	Spain (*N* = 67)	UK (*N* = 61)
Age at 1LM (index), *n* (%)						
18–20	5 (1.55)	0 (0.00)	1 (1.67)	1 (1.37)	3 (4.48)	0 (0.00)
21–45	5 (1.55)	0 (0.00)	1 (1.67)	1 (1.37)	2 (2.99)	1 (1.64)
46–65	127 (39.44)	28 (45.90)	17 (28.33)	33 (45.21)	25 (37.31)	24 (39.34)
66–75	147 (45.65)	30 (49.18)	31 (51.67)	27 (36.99)	27 (40.30)	32 (52.46)
≥76	38 (11.80)	3 (4.92)	10 (16.67)	11 (15.07)	10 (14.93)	4 (6.56)
Index year, *n* (%)						
2016	1 (0.31)	0 (0.00)	0 (0.00)	0 (0.00)	1 (1.49)	0 (0.00)
2017	0 (0.00)	0 (0.00)	0 (0.00)	0 (0.00)	0 (0.00)	0 (0.00)
2018	2 (0.62)	0 (0.00)	2 (3.33)	0 (0.00)	0 (0.00)	0 (0.00)
2019	8 (2.48)	1 (1.64)	3 (5.00)	2 (2.74)	1 (1.49)	1 (1.64)
2020	108 (33.54)	18 (29.51)	20 (33.33)	22 (30.14)	25 (37.31)	23 (37.70)
2021	203 (63.04)	42 (68.85)	35 (58.33)	49 (67.12)	40 (59.70)	37 (60.66)
Year of initial NSCLC diagnosis, *n* (%)						
2015	1 (0.31)	0 (0.00)	0 (0.00)	0 (0.00)	1 (1.49)	0 (0.00)
2016	1 (0.31)	0 (0.00)	1 (1.67)	0 (0.00)	0 (0.00)	0 (0.00)
2017	2 (0.62)	0 (0.00)	1 (1.67)	1 (1.37)	0 (0.00)	0 (0.00)
2018	2 (0.62)	0 (0.00)	1 (1.67)	1 (1.37)	0 (0.00)	0 (0.00)
2019	24 (7.45)	2 (3.28)	5 (8.33)	7 (9.59)	3 (4.48)	7 (11.48)
2020	180 (55.90)	40 (65.57)	38 (63.33)	30 (41.10)	38 (56.72)	34 (55.74)
2021	110 (34.16)	19 (31.15)	14 (23.33)	34 (46.58)	24 (35.82)	19 (31.15)
Unknown/missing	2 (0.62)	0 (0.00)	0 (0.00)	0 (0.00)	1 (1.49)	1 (1.64)
Sex, *n* (%)						
Male	229 (71.12)	45 (73.77)	44 (73.33)	50 (68.49)	49 (73.13)	41 (67.21)
Female	93 (28.88)	16 (26.23)	16 (26.67)	23 (31.51)	18 (26.87)	20 (32.79)
Smoking status at 1LM, *n* (%)						
Smoker	98 (30.43)	23 (37.70)	30 (50.00)	22 (30.14)	14 (20.90)	9 (14.75)
Ex‐smoker	193 (59.94)	36 (59.02)	28 (46.67)	36 (49.32)	46 (68.66)	47 (77.05)
Never smoked	22 (6.83)	2 (3.28)	2 (3.33)	10 (13.70)	3 (4.48)	5 (8.20)
Unknown	9 (2.80)	0 (0.00)	0 (0.00)	5 (6.86)	4 (5.97)	0 (0.00)
Stage at 1LM, *n* (%)						
Advanced	19 (5.90)	3 (4.92)	1 (1.67)	5 (6.85)	5 (7.46)	5 (8.20)
Stage IIIB	0 (0.00)	0 (0.00)	0 (0.00)	0 (0.00)	0 (0.00)	0 (0.00)
Stage IIIC	19 (5.90)	3 (4.92)	1 (1.67)	5 (6.85)	5 (7.46)	5 (8.20)
Metastatic	303 (94.10)	58 (95.08)	59 (98.33)	68 (93.15)	62 (92.54)	56 (91.80)
Stage IVA	97 (30.12)	16 (26.23)	13 (21.67)	25 (34.25)	20 (29.85)	23 (37.70)
Stage IVB	206 (63.98)	42 (68.85)	46 (76.67)	43 (58.90)	42 (62.69)	33 (54.10)
Histology, *n* (%)						
Squamous	97 (30.12)	22 (36.07)	21 (35.00)	21 (28.77)	9 (13.43)	24 (39.34)
Nonsquamous	219 (68.01)	39 (63.93)	39 (65.00)	49 (67.12)	55 (82.09)	37 (60.66)
Mixed	6 (1.86)	0 (0.00)	0 (0.00)	3 (4.11)	3 (4.48)	0 (0.00)
CNS/brain metastasis at 1LM, *n* (%)						
Asymptomatic CNS/brain metastasis	18 (5.59)	5 (8.20)	1 (1.67)	4 (5.48)	5 (7.46)	3 (4.92)
No CNS/brain metastasis	288 (89.44)	56 (91.80)	58 (96.67)	65 (89.04)	58 (86.57)	51 (83.61)
CNS/brain metastasis status unknown	16 (4.97)	0 (0.00)	1 (1.67)	4 (5.48)	4 (5.97)	7 (11.48)
ECOG PS at 1LM, *n* (%)						
0	75 (23.29)	10 (16.39)	12 (20.00)	23 (31.51)	12 (17.91)	18 (29.51)
1	247 (76.71)	51 (83.61)	48 (80.00)	50 (68.49)	55 (82.09)	42 (70.49)
PD‐L1 TPS tested, *n* (%)	**170 (54.31)**	**35 (59.32)**	**35 (58.33)**	**33 (45.21)**	**36 (54.55)**	**31 (56.36)**
TPS negative (<1%)	34 (20.00)	6 (17.14)	6 (17.14)	8 (24.24)	9 (25.00)	5 (16.13)
TPS 1%–49%	108 (63.53)	24 (68.57)	27 (77.14)	19 (57.58)	19 (52.78)	19 (61.29)
TPS ≥ 50%	28 (16.47)	5 (14.29)	2 (5.71)	6 (18.18)	8 (22.22)	7 (22.58)
Mortality status, *n* (%)						
Living	166 (51.55)	31 (50.82)	32 (53.33)	38 (52.05)	34 (50.75)	31 (50.82)
Deceased	156 (48.45)	30 (49.18)	28 (46.67)	35 (47.95)	33 (49.25)	30 (49.18)
Primary cause of death, *n* (%)						
NSCLC	116 (74.36)	23 (76.67)	24 (85.71)	26 (74.29)	24 (72.73)	19 (63.33)
Secondary comorbidities associated with the malignancy	12 (7.69)	4 (13.33)	2 (7.14)	3 (8.57)	2 (6.06)	1 (3.33)
COVID‐19	22 (14.10)	1 (3.33)	2 (7.14)	6 (17.14)	6 (18.18)	7 (23.33)
Other	6 (3.85)	2 (6.67)	0 (0.00)	0 (0.00)	1 (3.03)	3 (10.00)
Treatment funding of 1LM, *n* (%)						
Private healthcare	11 (3.42)	0 (0.00)	7 (11.67)	0 (0.00)	3 (4.48)	1 (1.64)
Public healthcare	304 (94.41)	60 (98.36)	53 (88.33)	73 (100.00)	64 (95.52)	54 (88.52)
Patient funding	0 (0.00)	0 (0.00)	0 (0.00)	0 (0.00)	0 (0.00)	0 (0.00)
Cancer drug fund (UK only)	6 (1.86)	0 (0.00)	0 (0.00)	0 (0.00)	0 (0.00)	6 (9.84)
Unknown	1 (0.31)	1 (1.64)	0 (0.00)	0 (0.00)	0 (0.00)	0 (0.00)

Bold indicates significance testing is not available.

Abbreviations: 1LM, first‐line maintenance; CNS, central nervous system; ECOG PS, Eastern Cooperative Oncology Group performance status; NSCLC, non‐small cell lung cancer; PD‐L1, programmed death‐ligand 1; TPS, tumor proportion score.

Most (68%) tumors were of nonsquamous histology, with the largest proportion observed in Spain (82%). Patients with squamous histology were almost equally likely to be smokers (48%) or ex‐smokers (46%), while patients with nonsquamous histology were more likely to be ex‐smokers (66%; Table S1). Of the 54% of patients with a PD‐L1 TPS result, most tumors (64%) had a PD‐L1 TPS of 1–49%, with Germany having the largest proportion (77%). Overall, the ECOG PS was 1 for 77% and 0 for the remaining 23%. Higher proportions of patients with an ECOG PS of 1 were observed in France, Germany, and Spain (80%–84%) compared with Italy or the UK (68%–70%). Similar proportions of patients were alive versus deceased, NSCLC being the primary cause of death. Treatment for 94% of patients was funded by public healthcare (Table 1).

### Physician characteristics

In total, 84 physicians completed the patient chart reviews (Table S2). Most physicians (83%) were clinical/medical oncologists. Practice settings were consistent across the EU5; more than half of physicians practicing in teaching/academic/research hospitals (52%). The majority of physician practice time was hospital‐based, except for Germany (85% office‐based). Overall, the included physicians had seen a median of 120 patients with NSCLC in the prior 12 months, the highest being in the UK (177 patients).

### Treatment patterns

#### 1L induction therapy

**FIGURE 1:**
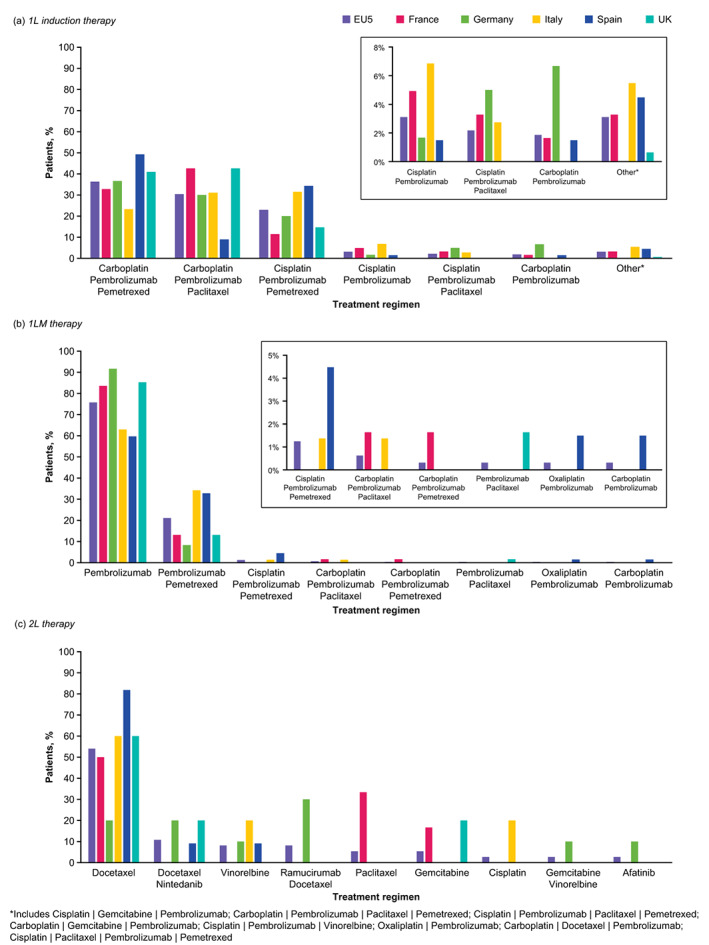
Treatment regimens used in (a) 1L induction, (b) 1LM and (c) 2L therapy in the EU5. *Includes Cisplatin + Gemcitabine + Pembrolizumab; Carboplatin + Pembrolizumab + Paclitaxel + Pemetrexed; Cisplatin + Pembrolizumab + Paclitaxel + Pemetrexed; Carboplatin + Gemcitabine + Pembrolizumab; Cisplatin + Pembrolizumab + Vinorelbine; Oxaliplatin + Pembrolizumab; Carboplatin + Docetaxel + Pembrolizumab; Cisplatin + Paclitaxel + Pembrolizumab + Pemetrexed. 1L, first line; 1LM, first‐line maintenance; 2L, second‐line.

The three most common 1L induction treatment regimens were carboplatin + pembrolizumab + pemetrexed (36%; most common in Germany and Spain); carboplatin + pembrolizumab + paclitaxel (30%; most common in France and the UK), and cisplatin + pembrolizumab + pemetrexed (23%; most common in Italy) (Figure 1; Table S3). Patients with nonsquamous NSCLC predominantly received pemetrexed‐containing 1L regimens, with carboplatin + pembrolizumab + pemetrexed the most commonly used regimen (49%; Table S4). Most patients with squamous histology (67%) received carboplatin + pembrolizumab + paclitaxel (Table S4).

Patients with a PD‐L1 TPS ≥50% were more commonly treated with carboplatin + pembrolizumab + pemetrexed (57%) than carboplatin + pembrolizumab + paclitaxel (32%); however, these two regimens were relatively balanced across patients with a TPS <1% (38% and 35%, respectively) and those with a TPS 1–49% (31% and 34%, respectively; Table S4).

#### 1LM therapy

Two treatment regimens accounted for 97% of 1LM regimens; pembrolizumab monotherapy (EU5, 76%; France, 84%; Germany, 92%; Italy, 63%; Spain, 60%; and UK, 43%) and pembrolizumab + pemetrexed (EU5, 21%; France, 13%; Germany, 8%; Italy, 34%; Spain, 33%; and UK, 13%; Figure 1). Pembrolizumab monotherapy was the most common 1LM regimen regardless of tumor histology or PD‐L1 status (Figure S2; Table S4).

#### 2L therapy

Only 11% of patients progressed to 2L therapy (France, *n* = 6; Germany, *n* = 10; Italy, *n* = 5; Spain, *n* = 11; and UK, *n* = 5). Among those patients, docetaxel monotherapy was the most used treatment (54%) (Figure 1) and was the top regimen for four of the five countries (France, 50%; Italy, 60%; Spain, 82%; and UK, 60%), with docetaxel + ramucirumab being the top regimen (30%) in Germany (Table S3).

#### 3L therapy

Only two patients progressed to 3L therapy (Tables S3 and S4), therefore 3L treatment patterns cannot be evaluated.

### Treatment outcomes

#### Duration of treatment

**FIGURE 2:**
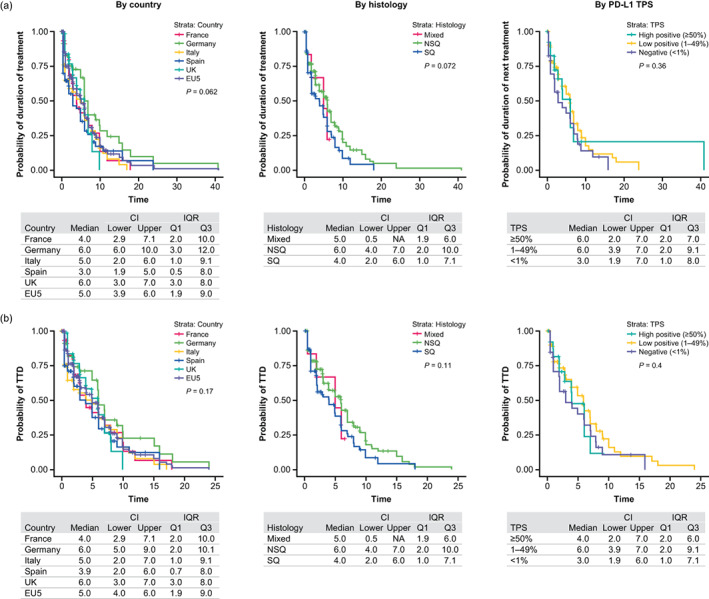
(a) Duration of 1LM treatment and (b) time to discontinuation of 1LM by country, histology, and PD‐L1 TPS. 1LM, first‐line maintenance; CI, confidence interval; IQR, interquartile range; NA, not applicable; NSQ, nonsquamous; PD‐L1, programmed death‐ligand 1; Q, quartile; SQ, squamous; TPS, tumor proportion score; TTD, time to discontinuation.

The overall median DOT for 1LM therapy was 5 months, with values ranging from 3 months in Spain to 6 months in Germany (Figure 2a, Table S5). A significant difference was observed in the median DOT for patients who achieved a CR/PR to 1L induction versus those with SD (6 months vs. 2 months; *p* < 0.0001) as well as between patients who did not have asymptomatic CNS/brain metastases at index versus those who did or whose status was unknown (6 months vs. 2 months vs. 3 months; *p* = 0.0003; Table S6). Median 1LM DOT for patients with squamous and nonsquamous histology was 4 and 6 months, respectively (Figure 2; Table S7). Patients with a negative TPS (<1%) had a shorter median 1LM DOT (3 months) than patients with a TPS of 1–49% or ≥50% (6 months for both; Figure 2; Table S7); however, this was not statistically significant.

#### Time to discontinuation

The overall median TTD of 1LM was 5 months, ranging from 4 months in Spain to 6 months in Germany and the UK (Figure 2b; Table S5). While not statistically significant, the median TTD of 1LM was numerically shorter for patients with squamous histology (4 months) versus those with nonsquamous histology (6 months, Table S7). Stratification of the nonsquamous subgroup by the presence or absence of pemetrexed in 1LM regimens revealed no difference in TTD of 1LM (Table S8). However, as with DOT, significant differences were observed between the median TTD of 1LM for patients who achieved a CR/PR to 1L induction versus those with SD (6 months vs. 2 months; *p* < 0.001) and between patients who did not have asymptomatic CNS/brain metastases at index date versus those who did, or those for whom status was unknown (6 months vs. 2 months vs. 2 months; *p* = 0.00018, Table S6). Median TTD was shorter for patients with PD‐L1 TPS <1% (3 months) compared with those with TPS 1%–49% (6 months); however, this was not statistically significant (Table S7). The most common reasons for 1LM treatment discontinuation were death (37%), distant (CNS/brain and extracranial) progression or relapse (24%), and local progression or relapse (23%) (Table S9). These trends were the same across the EU5; however, poor ECOG PS was also a key driver of treatment discontinuation in France (19%), and withdrawal of consent/patient's choice was the third most common reason for 1LM discontinuation in Italy (16%) over local progression or relapse (13%). The most common reasons for discontinuation were the same regardless of histology and in patients with PD‐L1 TPS <50%; however, for patients with a TPS ≥50%, local progression or relapse was not one of the most common reasons for treatment discontinuation (Table S10).

#### Real‐world time to next treatment

**FIGURE 3:**
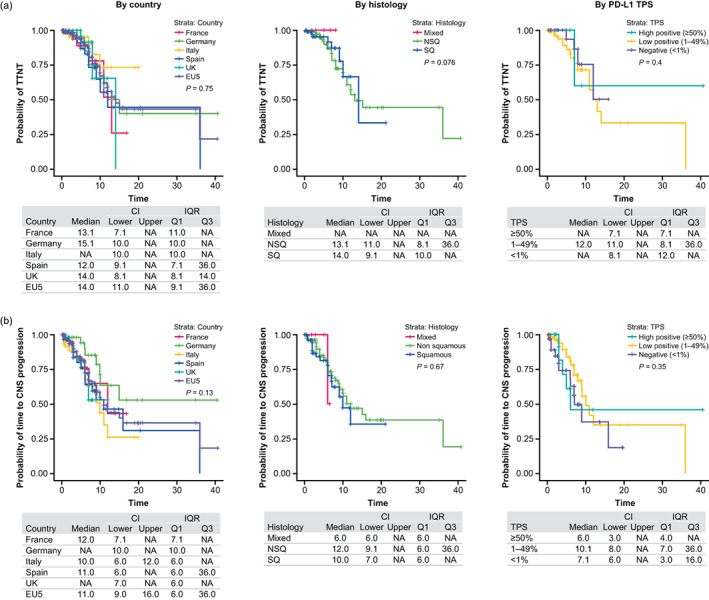
(a) Real‐world time to next treatment from 1LM to 2L and (b) time to CNS/brain progression by country, histology and PD‐L1 TPS from start of 1LM. 1LM, first‐line maintenance; 2L, second‐line; CI, confidence interval; CNS, central nervous system; IQR, interquartile range; NA, not applicable; NSQ, nonsquamous; PD‐L1, programmed death‐ligand 1; Q, quartile; SQ, squamous; TPS, tumor proportion score; TTNT, time to next treatment.

The overall median rwTTNT from 1LM to 2L was 14 months, ranging from 12 months in Spain to 15 months in Germany (Figure 3a; Table S5). Median rwTTNT from 1LM to 2L was similar between patients with squamous and nonsquamous histology but could not be calculated for the PD‐L1 TPS <1% or ≥50% subgroups due to the numbers of censored patients (Table S7).

#### Time to CNS/brain progression from start of 1LM therapy

The overall median time to CNS/brain progression was 11 months from start of 1LM (Figure 3b). Patients with asymptomatic CNS/brain metastases at index (*n* = 18) had significantly shorter (*p* = 0.002) median time to CNS/brain progression compared with those without CNS/brain metastases at index (5 months [CI: 3.0–NA] vs. 12 months [CI: 9.1–36.0], respectively). Patients with mixed histology had a nonsignificant shorter time to progression than those with squamous or nonsquamous histology (6 months [CI: 6–NA] vs. 10 months [CI: 7–NA] and 12 months [CI: 9–NA], respectively). Time to CNS/brain progression was significantly longer (*p* < 0.0001) in patients who achieved CR/PR to 1L induction therapy versus those who achieved SD (12 months [CI: 10–36] vs. 6 months [CI: 4–NA], respectively).

#### Treatment response rates

**FIGURE 4:**
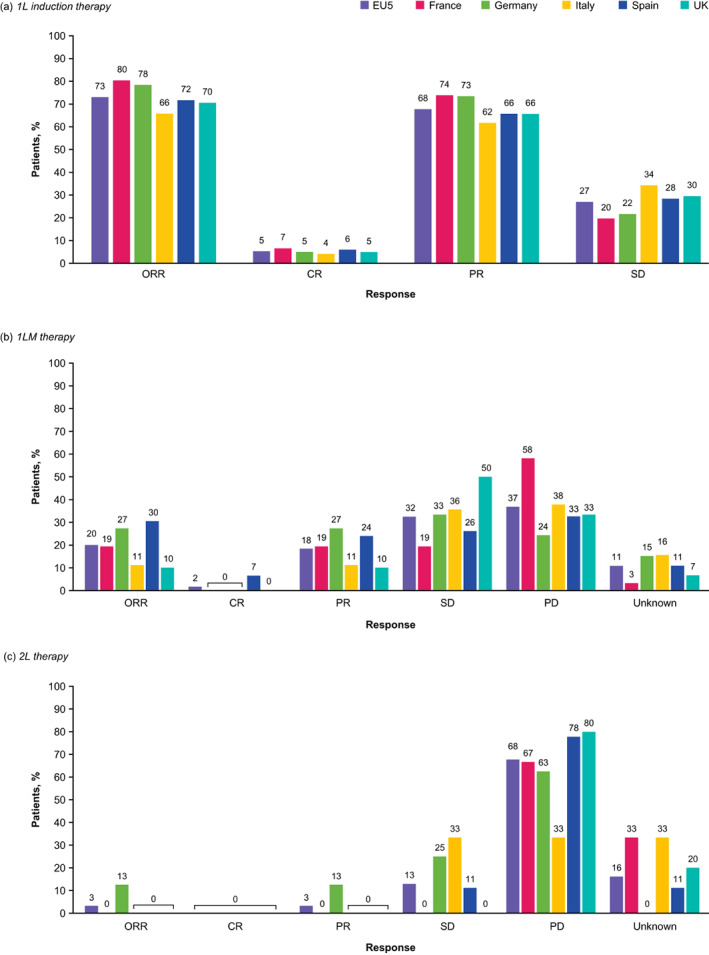
Treatment response rates in (a) 1L induction, (b) 1LM, and (c) 2L treatment in the EU5. 1L, first‐line; 1LM, first‐line maintenance; 2L, second‐line; CR, complete response; ORR, objective response rate; PD, progressive disease; PR, partial response; SD, stable disease.

The ORR in the EU5 dropped from 73% in 1L induction therapy to 20% in 1LM therapy, with 2%, 18%, and 32% of patients receiving 1LM achieving CR, PR, and SD, respectively (Figure 4). The median DOR for 1LM was 10 months for the EU5, ranging from 8 months in the UK to 12 months in Spain. Patients with squamous histology had a lower DOR to 1LM than those with nonsquamous histology (8 months vs. 11 months, respectively). DOR to 1LM was significantly longer (*p* < 0.0001) in patients without asymptomatic CNS/brain metastases at index (11 months [CI: 9–12]) than those with metastases (1 month [CI: 0.5–1.0]) and those with unknown status (5 months [CI: 4–NA]).

#### Progression‐free survival

**FIGURE 5:**
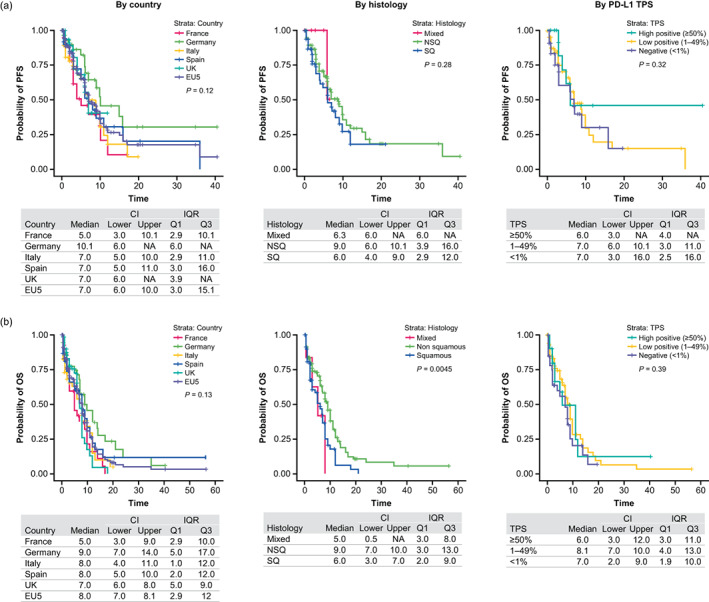
(a) Progression‐free survival* and (b) overall survival by country, histology, and PD‐L1 TPS. *Defined as the time (months) from start of 1LM therapy to the date of first documented progression or censored event. Progression‐free survival was based on documented progression alone; line advancement was not considered when determining progression. 1LM, first‐line maintenance; CI, confidence interval; IQR, interquartile range; NA, not applicable; NSQ, nonsquamous; OS, overall survival; PD‐L1, programmed death‐ligand 1; Q, quartile; SQ, squamous; TPS, tumor proportion score.

The overall median PFS in the EU5 was 7 months, ranging from 5 months (France) to 10 months (Germany; Figure 5). Patients with squamous and mixed histology had a shorter median PFS of 6 months each, compared with those with nonsquamous histology who had a median PFS of 9 months (*p* = 0.28). Median PFS was similar between patients with a PD‐L1 TPS ≥50% (6 months) and those with a PD‐L1 TPS of 1%–49% or <1% (7 months). Median PFS was significantly (*p* < 0.0001) longer for patients who achieved a PR/CR to 1L induction than for those with SD (10 months [CI: 7–10] vs. 4 months [CI: 3–6], respectively) and was also significantly longer for patients who did not have CNS/brain metastases at index than those who did (8 months [CI: 6–10] vs. 3 months [CI: 1–4]; *P* = 0.0019).

#### Overall survival

The median OS was 8 months in the EU5, ranging from 5 months (France) to 9 months (Germany; Figure 5b). Patients with squamous or mixed histology had a significantly lower median OS of 5 and 6 months, respectively, versus 9 months for patients with nonsquamous histology (*p* = 0.0045). No significant difference in OS was observed in patients stratified by PD‐L1 status. OS was significantly longer in patients who achieved a PR or CR to 1L induction therapy than those who achieved SD (9 vs. 4 months, respectively; *p* < 0.0001).

#### Exploratory outcomes

The overall median time to death from the start of 1LM therapy was 5 months (CI: 3–6). Patients with squamous or mixed histology had a nonsignificant shorter time to death versus those with nonsquamous histology (3 months [CI: 2–6] and 4 months [CI: 0.5–NA] vs. 6 months [CI: 3–7], respectively). The overall time to CNS/brain progression from the start of 1L induction therapy was 16 months (CI: 14–39).

## DISCUSSION

This was a retrospective study of treatment patterns and clinical outcomes in patients with advanced/metastatic NSCLC without known driver alterations who had received pembrolizumab as part of 1L induction and 1LM therapy in the EU5. This patient population is of interest given the challenge of treating patients without targetable gene alterations.^1^

Overall, carboplatin + pembrolizumab + pemetrexed was the most commonly used 1L regimen; however, this may have been skewed by high usages in Germany and Spain when compared with the other three countries. This is similar to retrospective data (August 2018–December 2019) from the US Flatiron Health oncology database which showed that 75% of patients with metastatic NSCLC and no EGFR or ALK mutations received immunotherapy as 1L treatment, with carboplatin + pembrolizumab + pemetrexed and carboplatin + pembrolizumab + paclitaxel being the two most common 1L regimens.^4^ Across the EU5 countries in this study, 5% of patients achieved a CR and 68% achieved a PR to 1L induction, with an ORR of 73%; these proportions may not reflect the general advanced/metastatic NSCLC population given the minimum of SD following 1L induction inclusion criterion.

In this study, 1LM therapy was consistent across all 5 countries, with pembrolizumab monotherapy the most used regimen regardless of tumor histology or PD‐L1 status, and docetaxel monotherapy the most common 2L regimen in all but Germany, which had equal usage of docetaxel + nintedanib. Since the introduction of ICIs in 2018, there have been limited real‐world studies of 1LM treatment patterns in advanced/metastatic NSCLC; however, increased adoption of pembrolizumab‐based regimens and decreased use of pemetrexed‐based regimens, regardless of 1L therapy, has been reported,^5^ reflecting the 2018 update of ESMO guidelines.^2^ Similarly, a retrospective analysis of clinical data from China between September 2018 and May 2021 reported that for patients with nonsquamous NSCLC who received pemetrexed + platinum‐based chemotherapy as 1L treatment, 17.7% went on to receive ICI monotherapy as 1LM while 38.1% received ICIs + pemetrexed.^6^

The overall median PFS on 1LM was 7 months. The median PFS was higher in patients with nonsquamous than squamous histology and was similar to that reported in the KEYNOTE‐189 and KEYNOTE‐407 trials, which had similar populations but only excluded ALK and EGFR driver alterations.^7^, ^8^, ^9^ In the KEYNOTE‐189 5‐year follow‐up, patients with nonsquamous metastatic NSCLC who received pembrolizumab as part of 1L and 1LM treatment had a median PFS of 9 months,^9^ similar to the current study. In the KEYNOTE‐407 5‐year follow‐up, patients with squamous metastatic NSCLC who received pembrolizumab in combination with chemotherapy as 1L and 1LM had a median PFS of 8 months,^10^ compared with 6 months in the current study. Though median PFS was similar across the PD‐L1 TPS subgroups in the current study, both KEYNOTE‐189 and KEYNOTE‐407 reported pembrolizumab addition favored patients with a PD‐L1 TPS ≥50% over those with a PD‐1L TPS <1%.^7^, ^8^ In the retrospective study in China, patients with a PD‐L1 TPS <1% benefited more from the ICI + pemetrexed combination than ICI monotherapy (median PFS of 10.92 months vs. 6.83 months; *p* = 0.008), while median PFS in patients with 1%–49% or >50% PD‐L1 TPS was similar between those who received monotherapy and combination therapy.^6^ The same study reported that brain metastasis affected PFS,^6^ which supports our finding that patients with CNS/brain metastases had a significantly shorter PFS than those without. In the current study, the median time to CNS/brain progression was 11 months and OS was 8 months, both of which were longer for patients with nonsquamous versus squamous histology. Unsurprisingly, PFS, time to CNS/brain progression, and OS were longer in patients who had achieved a CR or PR with 1L induction therapy versus those with SD; however, the decreased response to treatment with subsequent LOTs reflects the increasing difficulty of treating advanced/metastatic NSCLC following previous LOTs failure and the poor patient survival rate.

Across the EU5, ~57% of patients were recorded to have discontinued 1LM therapy, death, CNS/brain progression/relapse, and local progression/relapse the most common reasons. This is supported by a real‐world study on patients with NSCLC receiving 1L or 1LM therapy, where the main reason for discontinuation was tumor progression.^11^ The same study also reported that discontinuation of the 1LM was predominantly made by the physician, as opposed to shared decision‐making or by the patient. In addition to disease progression, patients' reasons for 1LM discontinuation included the need for a treatment break, toxicity or side‐effects, and physical and emotional exhaustion.^11^

### Strengths and limitations

The patient chart review in this study provided a comprehensive treatment history from the time of NSCLC diagnosis. All participating physicians were experienced with the management of NSCLC and had been treating patients with advanced/metastatic NSCLC, receiving 1LM, within 12 months of data extraction. The study design enabled the acquisition of a large amount of data on multiple clinical outcomes, variables, and study‐specific patient populations and subgroups, making it available to physicians and researchers in a shorter time frame than a clinical trial. Trends identified in the data may help guide treatment decisions for various patient populations.

Some limitations exist, however, and while the inclusion of both alive and deceased patients allowed analysis of the clinical outcomes of OS and time to death, it may have introduced some bias, including a higher probability of capturing patients with more aggressive disease. However, the quota for the number of deceased patients was conservative and could have led to underestimations of the true proportion of deceased patients. PD‐L1 status would likely have influenced treatment decision‐making; however, sensitivity analyses were not conducted to control for this. Furthermore, certain analyses were limited by the low sample size; for example, as only two patients receiving 3L therapy were included in the study, it was not possible to identify treatment patterns in this subgroup. Additional study limitations include the potential for reporting bias given data was provided by eligible physicians who chose to participate in the study only, and that the study sample may not be representative of the advanced/metastatic NSCLC population of each country, in addition to the inherent limitations that come with retrospective studies, such as the handling of missing data.

In conclusion, this study provides detailed real‐world data on treatment patterns and clinical outcomes in patients with advanced/metastatic NSCLC in the EU5 and demonstrates notable differences across countries and patient subgroups. While 1L and 2L regimens differed between the five countries, pembrolizumab monotherapy was the most used 1LM regimen across the EU5. Improved survival outcomes were generally associated with response to 1L therapy, nonsquamous histology, and the absence of CNS/brain metastases; however, survival rates generally remain poor. This valuable insight into real‐world clinical practice highlights the need for efficacious treatments in this patient population and may aid future treatment decision‐making and clinical trial design.

## AUTHOR CONTRIBUTIONS

Conceptualization, Amine Aziez, Alexander Stojadinovic, Chi‐Chang Chen, and Fil Manuguid; Formal analysis, Amine Aziez, Alexander Slowley, Alexander Stojadinovic, Chi‐Chang Chen, Fil Manuguid, Jessica Chao, Jasjit K. Multani, Kelly F. Bell, Kelesitse Phiri, Marie Yasuda, Sheila Mpima, and Vicky Casey; Investigation, Chi‐Chang Chen, Fil Manuguid, Jasjit K. Multani, Marie Yasuda, Sheila Mpima, and Vicky Casey; Methodology, Amine Aziez, Alexander Stojadinovic, Chi‐Chang Chen, Fil Manuguid, Jessica Chao, Jasjit K. Multani, Marie Yasuda, Sheila Mpima, and Vicky Casey. Writing – original draft, all authors. Writing – Review and editing, all authors. All authors had access to the data in the study and take responsibility for the integrity of the data and the accuracy of the analysis.

## FUNDING INFORMATION

This study was conducted by IQVIA with funding support from GSK (GSK study 214794).

## CONFLICT OF INTEREST STATEMENT

AA and KP are employees of GSK. ASl, ASt, JC, and KFB are employees of, and hold stocks, in GSK. JKM, VC, SM, MY, CCC, and FM are employees of IQVIA. IQVIA was contracted by GSK to conduct this study.

## Supporting information


**Figure S1.** Timing of study measures for eligible patients.

**Figure S2.** Treatment regimens used in (a) 1LM by histology, (b) 2L therapy by histology, (c) 1LM by PD‐L1 TPS and (d) 2L therapy by PD‐L1 TPS.

**Table S1.** Patient characteristics by histology and PD‐L1 TPS.
**Table S2.** Physician characteristics.
**Table S3.** Individual treatments and treatment regimens per LOT, in the EU5 and by country.
**Table S4.** Treatment regimens per LOT by histology and PD‐L1 TPS.
**Table S5.** DOT, TTD, and rwTTNT for each LOT, in the EU5 and by country.
**Table S6.** DOT, TTD, and rwTTNT for each LOT by response to 1L induction and by CNS/brain metastases status (at index date).
**Table S7.** DOT, TTD, and rwTTNT for each LOT by histology and PD‐L1 TPS.
**Table S8.** DOT, TTD, and rwTTNT for each LOT in patients with non‐squamous histology, by use of pemetrexed in 1LM regimen.
**Table S9.** Reasons for LOT discontinuation in EU5 and by country.
**Table S10.** Reasons for LOT discontinuation by histology and PD‐L1 TPS.

## Data Availability

GSK makes available anonymized individual participant data and associated documents from interventional clinical studies that evaluate medicines, upon approval of proposals submitted to https://www.gsk-studyregister.com/en/. To access data for other types of GSK sponsored research, for study documents without patient‐level data, and for clinical studies not listed, please submit an enquiry via this website.
