# Supplementary figures and images for: Real‐world treatment patterns and clinical outcomes after introduction of immune checkpoint inhibitors: Results from a retrospective chart review of patients with advanced/metastatic non‐small cell lung cancer in the EU5
Source: Thorac Cancer. 2023 Aug 17;14(28):2846–58. doi: 10.1111/1759-7714.15069 (PMC10542458; doi:10.1111/1759-7714.15069)

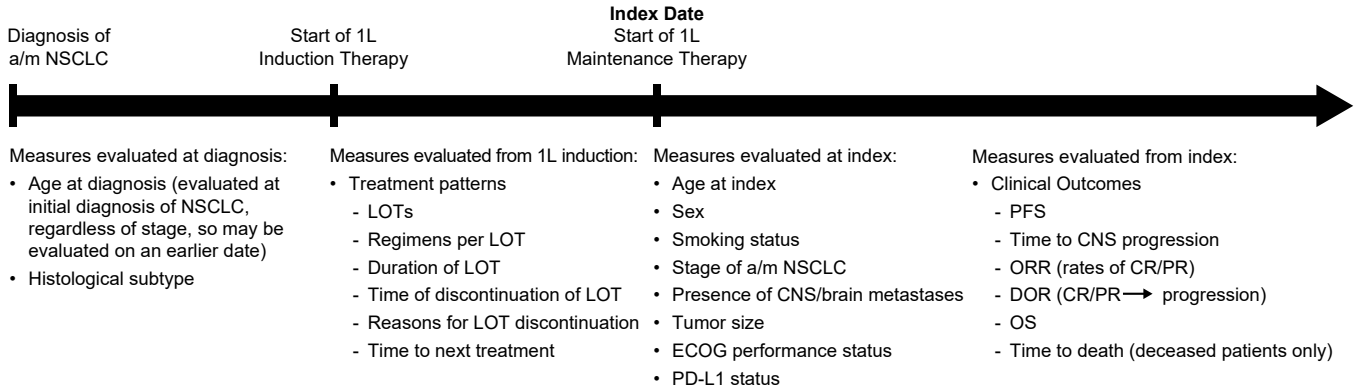

Supplement: Supplementary file 1 — Figure S1. Timing of study measures for eligible patients. [file TCA-14-2846-s002.pdf]

A. 1LM therapy by histology

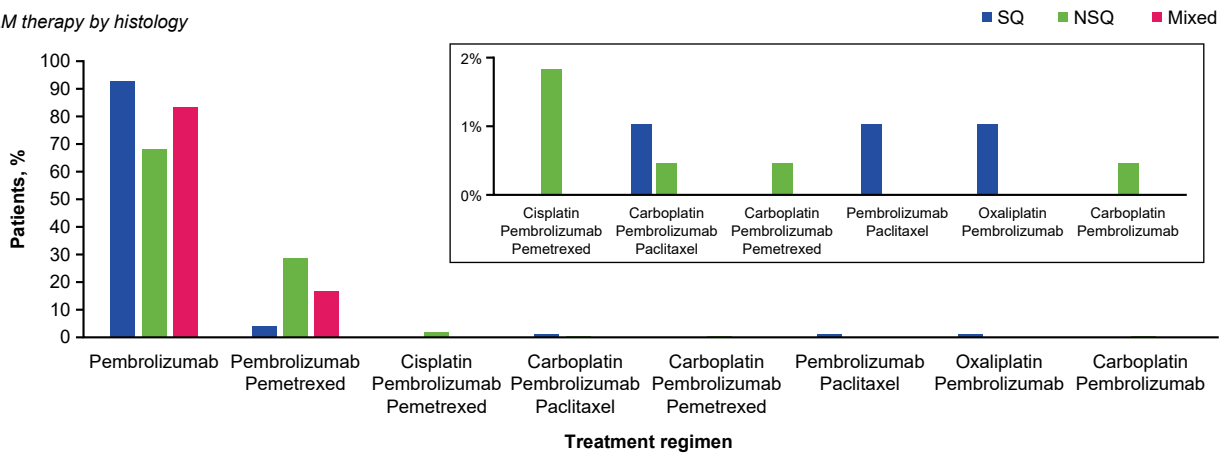

B. 2L therapy by histology

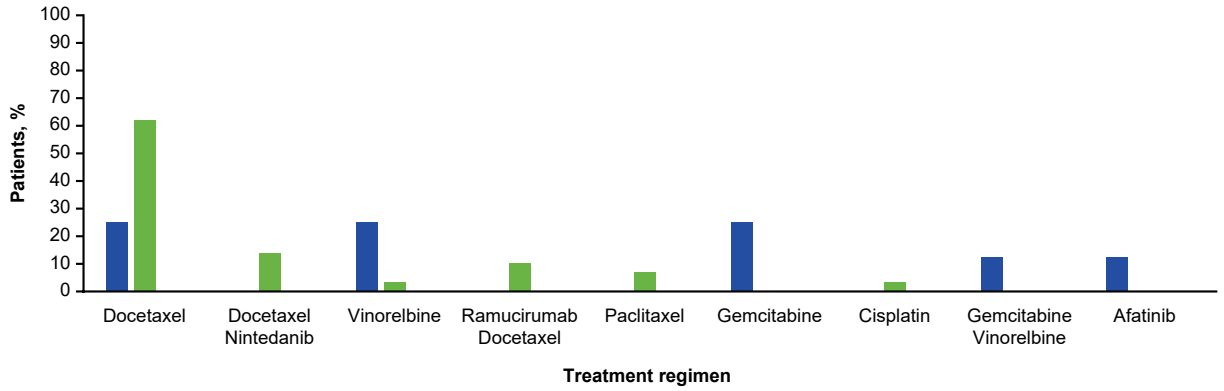

C. 1LM therapy by PD-L1 TPS

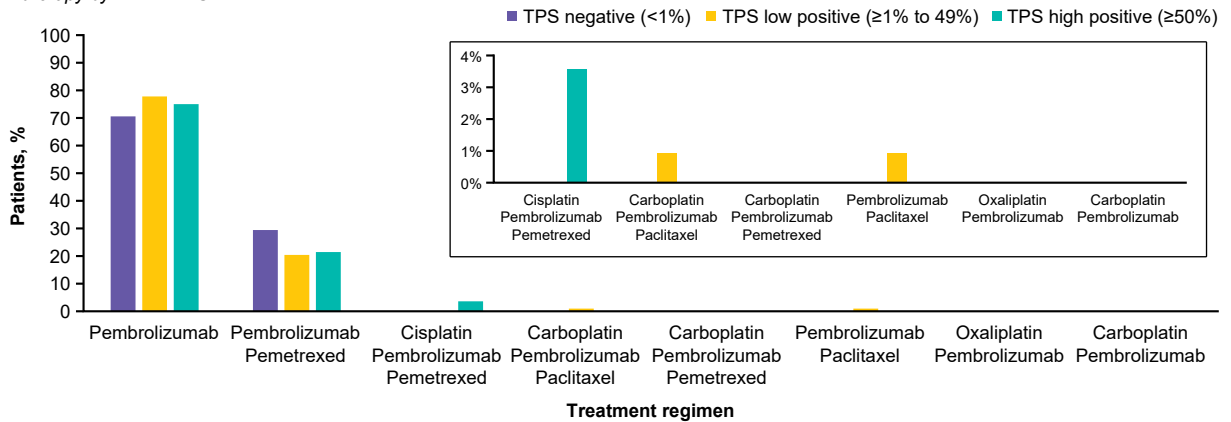

D. 2L therapy by PD-L1 TPS

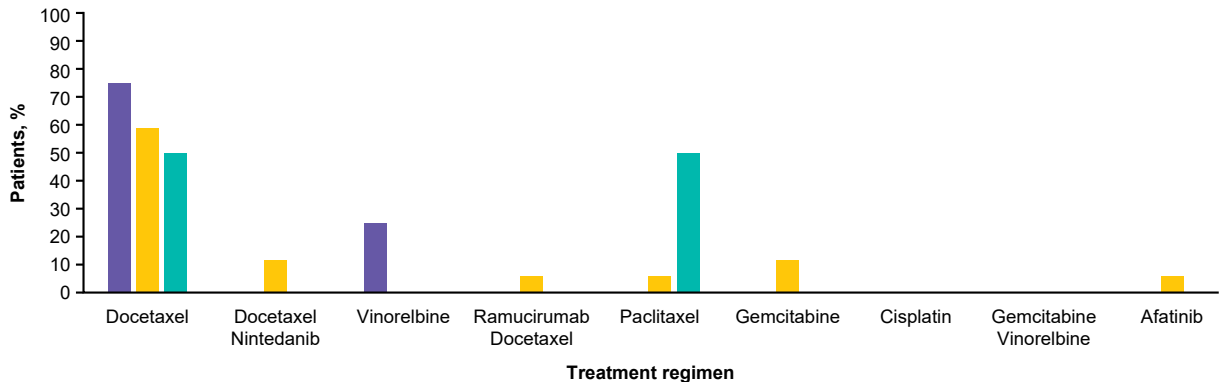

Supplement: Supplementary file 2 — Figure S2. Treatment regimens used in (a) 1LM by histology, (b) 2L therapy by histology, (c) 1LM by PD‐L1 TPS and (d) 2L therapy by PD‐L1 TPS. [file TCA-14-2846-s003.pdf]
