# Supplementary material for: Real‐world treatment patterns and clinical outcomes after introduction of immune checkpoint inhibitors: Results from a retrospective chart review of patients with advanced/metastatic non‐small cell lung cancer in the EU5
Source: Thorac Cancer. 2023 Aug 17;14(28):2846–58. doi: 10.1111/1759-7714.15069 (PMC10542458; doi:10.1111/1759-7714.15069)
Supplement: Supplementary file 3 — Table S1. Patient characteristics by histology and PD‐L1 TPS. Table S2. Physician characteristics. Table S3. Individual treatments and treatment regimens per LOT, in the EU5 and by country. Table S4. Treatment regimens per LOT by histology and PD‐L1 TPS. Table S5. DOT, TTD, and rwTTNT for each LOT, in the EU5 and by country. Table S6. DOT, TTD, and rwTTNT for each LOT by response to 1L induction and by CNS/brain metastases status (at index date). Table S7. DOT, TTD, and rwTTNT for each LOT by histology and PD‐L1 TPS. Table S8. DOT, TTD, and rwTTNT for each LOT in patients with non‐squamous histology, by use of pemetrexed in 1LM regimen. Table S9. Reasons for LOT discontinuation in EU5 and by country. Table S10. Reasons for LOT discontinuation by histology and PD‐L1 TPS. [file TCA-14-2846-s001.docx]

# Supplementary Tables

Supplementary **Table 1.** Patient characteristics by histology and PD-L1 TPS

|  | **Histology at diagnosis of advanced/metastatic NSCLC** | | | **PD-L1 status (by TPS) at index date** | | |
| --- | --- | --- | --- | --- | --- | --- |
|  | **Squamous (N=97)** | **Non-squamous (N=219)** | **Mixed (N=6)** | **TPS negative (<1%)  (N=34)** | **TPS  ≥1–49%  (N=108)** | **TPS ≥50%**  **(N=28)** |
| **Age at 1LM (index), n (%)** |  |  |  |  |  |  |
| **18–20** | 1 (1.03) | 4 (1.83) | 0 (0.00) | 1 (2.94) | 1 (0.93) | 0 (0.00) |
| **21–45** | 0 (0.00) | 5 (2.28) | 0 (0.00) | 2 (5.88) | 1 (0.93) | 1 (3.57) |
| **46–65** | 36 (37.11) | 87 (39.73) | 4 (66.67) | 12 (35.29) | 44 (40.74) | 12 (42.86) |
| **66–75** | 48 (49.48) | 99 (45.21) | 0 (0.00) | 13 (38.24) | 47 (43.52) | 13 (46.43) |
| **≥76** | 12 (12.37) | 24 (10.96) | 2 (33.33) | 6 (17.65) | 15 (13.89) | 2 (7.14) |
| **Index year, n (%)** |  |  |  |  |  |  |
| **2016** | 0 (0.00) | 1 (0.46) | 0 (0.00) | 0 (0.00) | 1 (0.93) | 0 (0.00) |
| **2017** | 0 (0.00) | 0 (0.00) | 0 (0.00) | 0 (0.00) | 0 (0.00) | 0 (0.00) |
| **2018** | 0 (0.00) | 2 (0.91) | 0 (0.00) | 0 (0.00) | 1 (0.93) | 1 (3.57) |
| **2019** | 2 (2.06) | 6 (2.74) | 0 (0.00) | 1 (2.94) | 4 (3.70) | 0 (0.00) |
| **2020** | 32 (32.99) | 73 (33.33) | 3 (50.00) | 18 (52.94) | 29 (26.85) | 6 (21.43) |
| **2021** | 63 (64.95) | 137 (62.56) | 3 (50.00) | 15 (44.12) | 73 (67.59) | 21 (75.00) |
| **Year of initial NSCLC diagnosis, n (%)** |  |  |  |  |  |  |
| **2015** | 0 (0.00) | 1 (0.46) | 0 (0.00) | 0 (0.00) | 1 (0.93) | 0 (0.00) |
| **2016** | 0 (0.00) | 1 (0.46) | 0 (0.00) | 0 (0.00) | 1 (0.93) | 0 (0.00) |
| **2017** | 0 (0.00) | 2 (0.91) | 0 (0.00) | 0 (0.00) | 0 (0.00) | 1 (3.57) |
| **2018** | 2 (2.06) | 0 (0.00) | 0 (0.00) | 0 (0.00) | 2 (1.85) | 0 (0.00) |
| **2019** | 5 (5.15) | 19 (8.68) | 0 (0.00) | 4 (11.76) | 6 (5.56) | 3 (10.71) |
| **2020** | 59 (80.82) | 116 (52.97) | 5 (83.33) | 20 (58.82) | 57 (52.78) | 15 (53.57) |
| **2021** | 31 (31.96) | 78 (35.62) | 1 (16.67) | 9 (26.47) | 41 (37.96) | 9 (32.14) |
| **Unknown/**  **missing** | 0 (0.00) | 2 (0.91) | 0 (0.00) | 1 (2.94) | 0 (0.00) | 0 (0.00) |
| **Sex, n (%)** |  |  |  |  |  |  |
| **Male** | 71 (73.20) | 154 (70.32) | 4 (66.67) | 20 (58.82) | 82 (75.93) | 21 (75.00) |
| **Female** | 26 (26.80) | 65 (29.68) | 2 (33.33) | 14 (41.18) | 26 (24.07) | 7 (25.00) |
| **Smoking status at 1LM, n (%)** |  |  |  |  |  |  |
| **Smoker** | 47 (48.45) | 50 (22.83) | 1 (16.67) | 12 (35.29) | 35 (32.41) | 10 (35.71) |
| **Ex-smoker** | 45 (46.39) | 144 (65.75) | 4 (66.67) | 20 (58.82) | 66 (61.11) | 15 (53.57) |
| **Never smoked** | 4 (4.12) | 18 (8.22) | 0 (0.00) | 2 (5.88) | 6 (5.56) | 2 (7.14) |
| **Unknown** | 1 (1.03) | 7 (3.20) | 1 (16.67) | 0 (0.00) | 1 (0.93) | 1 (3.57) |
| **Stage at 1LM, n (%)** |  |  |  |  |  |  |
| **Advanced** | 3 (3.09) | 15 (6.85) | 1 (16.67) | 2 (5.88) | 8 (7.41) | 1 (3.57) |
| **Stage IIIB** | 0 (0.00) | 0 (0.00) | 0 (0.00) | 0 (0.00) | 0 (0.00) | 0 (0.00) |
| **Stage IIIC** | 3 (3.09) | 15 (6.85) | 1 (16.67) | 2 (5.88) | 8 (7.41) | 1 (3.57) |
| **Metastatic** | 94 (96.91) | 204 (93.15) | 5 (83.33) | 32 (94.12) | 100 (92.59) | 27 (96.43) |
| **Stage IVA** | 32 (32.99) | 64 (29.22) | 1 (16.67) | 7 (20.59) | 34 (31.48) | 13 (46.43) |
| **Stage IVB** | 62 (63.92) | 140 (63.93) | 4 (66.67) | 25 (73.53) | 66 (61.11) | 14 (50.00) |
| **Histology, n (%)** |  |  |  |  |  |  |
| **Squamous** |  |  |  | 11 (32.35) | 32 (29.63) | 7 (25.00) |
| **Non-squamous** |  |  |  | 23 (67.65) | 76 (70.37) | 20 (71.43) |
| **Mixed** |  |  |  | 0 (0.00) | 0 (0.00) | 1 (3.57) |
| **CNS/brain metastasis at 1LM, n (%)** |  |  |  |  |  |  |
| **Asymptomatic CNS/brain metastasis** | 8 (8.25) | 9 (4.11) | 1 (16.67) | 2 (5.88) | 4 (3.70) | 3 (10.71) |
| **No CNS/brain metastasis** | 85 (87.63) | 199 (90.87) | 4 (66.67) | 31 (91.18) | 101 (93.52) | 23 (82.14) |
| **CNS/brain metastasis status unknown** | 4 (4.12) | 11 (5.02) | 1 (16.67) | 1 (2.94) | 3 (2.78) | 2 (7.14) |
| **ECOG PS at 1LM, n (%)** |  |  |  |  |  |  |
| **0** | 22 (22.68) | 51 (23.29) | 2 (33.33) | 10 (29.41) | 26 (24.07) | 7 (25.00) |
| **1** | 75 (77.32) | 168 (76.71) | 4 (66.67) | 24 (70.59) | 82 (75.93) | 21 (75.00) |
| **PD-L1 status, n (%)** |  |  |  |  |  |  |
| **TPS negative (<1%)** | 11 (22.00) | 23 (19.33) | 0 (0.00) |  |  |  |
| **TPS 1–49%** | 32 (64.00) | 76 (63.87) | 0 (0.00) |  |  |  |
| **TPS ≥50%** | 7 (14.00) | 20 (16.81) | 1 (100.00) |  |  |  |
| **Mortality status, n (%)** |  |  |  |  |  |  |
| **Living** | 42 (43.40) | 122 (55.71) | 2 (33.33) | 11 (32.35) | 61 (56.48) | 17 (60.71) |
| **Deceased** | 55 (56.70) | 97 (44.29) | 4 (66.67) | 23 (67.65) | 47 (42.52) | 11 (39.29) |
| **Primary cause of death, n (%)** |  |  |  |  |  |  |
| **NSCLC** | 41 (74.55) | 74 (76.29) | 1 (25.00) | 20 (86.96) | 32 (68.09) | 7 (63.64) |
| **Secondary comorbidities associated with the malignancy** | 3 (5.45) | 9 (9.28) | 0 (0.00) | 0 (0.00) | 7 (14.89) | 1 (9.09) |
| **COVID-19** | 9 (16.36) | 10 (10.31) | 3 (75.00) | 2 (8.70) | 6 (12.77) | 2 (18.18) |
| **Other** | 2 (3.64) | 4 (4.12) | 0 (0.00) | 1 (4.35) | 2 (4.26) | 1 (9.09) |
| **Treatment funding of 1LM, n (%)** |  |  |  |  |  |  |
| **Private healthcare** | 6 (6.19) | 5 (2.28) | 0 (0.00) | 0 (0.00) | 3 (2.78) | 0 (0.00) |
| **Public healthcare** | 88 (90.72) | 210 (95.89) | 6 (100.00) | 34 (100.00) | 102 (94.44) | 26 (92.86) |
| **Patient funding** | 0 (0.00) | 0 (0.00) | 0 (0.00) | 0 (0.00) | 0 (0.00) | 0 (0.00) |
| **Cancer drug fund (UK only)** | 3 (3.09) | 3 (1.37) | 0 (0.00) | 0 (0.00) | 3 (2.78) | 2 (7.14) |
| **Unknown** | 0 (0.00) | 1 (0.46) | 0 (0.00) | 0 (0.00) | 0 (0.00) | 0 (0.00) |

1LM, first-line maintenance; CNS, central nervous system; ECOG PS, Eastern Oncology Cooperative Group performance status; NSCLC, non-small cell lung cancer; PD-L1, programmed death-ligand 1; TPS, tumor proportion score

**Supplementary Table 2.** Physician characteristics

|  | **EU5  (N=322)** | **France (N=61)** | **Germany (N=60)** | **Italy  (N=73)** | **Spain  (N=67)** | **UK**  **(N=61)** |
| --- | --- | --- | --- | --- | --- | --- |
| **Unique treating physicians, n** |  |  |  |  |  |  |
| **Number of unique treating physicians** | 84 | 16 | 16 | 19 | 17 | 16 |
| **Physician specialty, n (%)** |  |  |  |  |  |  |
| **Clinical/medical oncologist** | 70 (83.4) | 9 (56.3) | 9 (56.3) | 19 (100.0) | 17 (100.0) | 16 (100.0) |
| **Pulmonologist** | 14 (16.7) | 7 (43.8) | 7 (43.8) | 0 (0.0) | 0 (0.0) | 0 (0.0) |
| **Time since specializing after board certification, n (%)** |  |  |  |  |  |  |
| **1–14 years** | 40 (47.6) | 3 (18.8) | 3 (18.8) | 16 (84.2) | 11 (64.7) | 7 (43.8) |
| **15–35 years** | 44 (52.4) | 13 (81.3) | 13 (81.3) | 3 (15.8) | 6 (35.3) | 9 (56.3) |
| **Percentage of practice time, per setting type in the last year, median** |  |  |  |  |  |  |
| **Hospital** | 92.5 | 100 | 10 | 90 | 100 | 80 |
| **Office** | 0 | 0 | 85 | 0 | 0 | 5 |
| **Virtual** | 0 | 0 | 0 | 0 | 0 | 10 |
| **Private or public hospital setting, n (%)** |  |  |  |  |  |  |
| **Cancer specialist hospital** | 16 (19.0) | 2 (12.5) | 1 (6.3) | 7 (36.8) | 2 (11.8) | 4 (25.0) |
| **Teaching/academic/research hospital** | 44 (52.4) | 9 (56.3) | 7 (43.8) | 8 (42.1) | 8 (47.1) | 12 (75.0) |
| **Secondary/general hospital** | 12 (14.3) | 5 (31.3) | 0 (0.0) | 4 (21.1) | 3 (17.6) | 0 (0.0) |
| **Tertiary hospital** | 4 (4.8) | 0 (0.0) | 0 (0.0) | 0 (0.0) | 4 (23.5) | 0 (0.0) |
| **Other (office-based; Germany)** | 8 (9.5) | 0 (0.0) | 8 (50.0) | 0 (0.0) | 0 (0.0) | 0 (0.0) |
| **Percentage of time devoted to seeing and treating patients last year** |  |  |  |  |  |  |
| **Median** | 90.0 | 92.5 | 87.5 | 90.0 | 90.0 | 85.0 |
| **Number of patients with NSCLC seen in the last 12 months** |  |  |  |  |  |  |
| **Median** | 120.0 | 100.0 | 87.5 | 150.0 | 150.0 | 177.5 |

NSCLC, non-small cell lung cancer

Supplementary **Table 3.** Individual treatments and treatment regimens per LOT, in the EU5 and by country

|  | **EU5  (N=322)** | **France (N=61)** | **Germany (N=60)** | **Italy  (N=73)** | **Spain  (N=67)** | **UK**  **(N=61)** |
| --- | --- | --- | --- | --- | --- | --- |
| **1L induction treatments received, n (%)** | **322 (100.00)** | **61 (100.00)** | **60 (100.00)** | **73 (100.00)** | **67 (100.00)** | **61 (100.00)** |
| **Pembrolizumab** | 322 (100.00) | 61 (100.00) | 60 (100.00) | 73 (100.00) | 67 (100.00) | 61 (100.00) |
| **Carboplatin** | 224 (69.57) | 48 (78.69) | 44 (73.33) | 40 (54.79) | 41 (61.19) | 51 (83.61) |
| **Pemetrexed** | 194 (60.25) | 28 (45.90) | 34 (56.67) | 43 (58.90) | 56 (83.58) | 34 (55.74) |
| **Paclitaxel** | 108 (33.54) | 27 (44.26) | 21 (35.00) | 27 (36.99) | 6 (8.96) | 26 (42.62) |
| **Cisplatin** | 97 (30.12) | 13 (21.31) | 16 (26.67) | 33 (45.21) | 25 (37.31) | 10 (16.39) |
| **Gemcitabine** | 4 (1.24) | 1 (1.64) | 0 (0.00) | 1 (1.37) | 2 (2.99) | 0 (0.00) |
| **Oxaliplatin** | 1 (0.31) | 0 (0.00) | 0 (0.00) | 0 (0.00) | 1 (1.49) | 0 (0.00) |
| **Vinorelbine** | 1 (0.31) | 0 (0.00) | 0 (0.00) | 0 (0.00) | 0 (0.00) | 1 (1.64) |
| **Docetaxel** | 1 (0.31) | 1 (1.64) | 0 (0.00) | 0 (0.00) | 0 (0.00) | 0 (0.00) |
| **1L induction regimen received, n (%)** | **322 (100.00)** | **61 (100.00)** | **60 (100.00)** | **73 (100.00)** | **67 (100.00)** | **61 (100.00)** |
| **Carboplatin \| Pembrolizumab \| Pemetrexed** | 117 (36.34) | 20 (32.79) | 22 (36.67) | 17 (23.29) | 33 (49.25) | 25 (40.98) |
| **Carboplatin \| Pembrolizumab \| Paclitaxel** | 98 (30.43) | 26 (42.62) | 18 (30.00) | 22 (30.14) | 6 (8.96) | 26 (42.62) |
| **Cisplatin \| Pembrolizumab \| Pemetrexed** | 74 (22.98) | 7 (11.48) | 12 (20.00) | 23 (31.51) | 23 (34.33) | 9 (14.75) |
| **Cisplatin \| Pembrolizumab** | 10 (3.11) | 3 (4.92) | 1 (1.67) | 5 (6.85) | 1 (1.49) | 0 (0.00) |
| **Cisplatin \| Pembrolizumab \| Paclitaxel** | 7 (2.17) | 2 (3.28) | 3 (5.00) | 2 (2.74) | 0 (0.00) | 0 (0.00) |
| **Carboplatin \| Pembrolizumab** | 6 (1.86) | 1 (1.64) | 4 (6.67) | 0 (0.00) | 1 (1.49) | 0 (0.00) |
| **Other*** | 10 (3.11) | 2 (3.28) | 0 (0.00) | 4 (5.48) | 3 (4.48) | 1 (0.64) |
| **1LM treatments, n (%)** | **322 (100.00)** | **61 (100.00)** | **60 (100.00)** | **73 (100.00)** | **67 (100.00)** | **61 (100.00)** |
| **Pembrolizumab** | 322 (100.00) | 61 (100.00) | 60 (100.00) | 73 (100.00) | 67 (100.00) | 61 (100.00) |
| **Pemetrexed** | 73 (22.67) | 9 (14.75) | 5 (8.33) | 26 (35.62) | 25 (37.31) | 8 (13.11) |
| **Cisplatin** | 4 (1.24) | 0 (0.00) | 0 (0.00) | 1 (1.37) | 3 (4.48) | 0 (0.00) |
| **Carboplatin** | 4 (1.24) | 2 (3.28) | 0 (0.00) | 1 (1.37) | 1 (1.49) | 0 (0.00) |
| **Paclitaxel** | 3 (0.93) | 1 (1.64) | 0 (0.00) | 1 (1.37) | 0 (0.00) | 1 (1.64) |
| **Oxaliplatin** | 1 (0.31) | 0 (0.00) | 0 (0.00) | 0 (0.00) | 1 (1.49) | 0 (0.00) |
| **1LM regimens, n (%)** | **322 (100.00)** | **61 (100.00)** | **60 (100.00)** | **73 (100.00)** | **67 (100.00)** | **61 (100.00)** |
| **Pembrolizumab** | 244 (75.78) | 51 (83.61) | 55 (91.67) | 46 (63.01) | 40 (59.70) | 52 (85.25) |
| **Pembrolizumab \| Pemetrexed** | 68 (21.12) | 8 (13.11) | 5 (8.33) | 25 (34.25) | 22 (32.84) | 8 (13.11) |
| **Cisplatin \| Pembrolizumab \| Pemetrexed** | 4 (1.24) | 0 (0.00) | 0 (0.00) | 1 (1.37) | 3 (4.48) | 0 (0.00) |
| **Carboplatin \| Pembrolizumab \| Paclitaxel** | 2 (0.62) | 1 (1.64) | 0 (0.00) | 1 (1.37) | 0 (0.00) | 0 (0.00) |
| **Carboplatin \| Pembrolizumab \| Pemetrexed** | 1 (0.31) | 1 (1.64) | 0 (0.00) | 0 (0.00) | 0 (0.00) | 0 (0.00) |
| **Pembrolizumab \| Paclitaxel** | 1 (0.31) | 0 (0.00) | 0 (0.00) | 0 (0.00) | 0 (0.00) | 1 (1.64) |
| **Oxaliplatin \| Pembrolizumab** | 1 (0.31) | 0 (0.00) | 0 (0.00) | 0 (0.00) | 1 (1.49) | 0 (0.00) |
| **Carboplatin \| Pembrolizumab** | 1 (0.31) | 0 (0.00) | 0 (0.00) | 0 (0.00) | 1 (1.49) | 0 (0.00) |
| **2L treatments, n (%)** | **37 (11.00)** | **6 (10.00)** | **10 (17.00)** | **5 (7.00)** | **11 (16.00)** | **5 (8.00)** |
| **Docetaxel** | 27 (72.97) | 3 (50.00) | 7 (70.00) | 3 (60.00) | 10 (90.91) | 4 (80.00) |
| **Vinorelbine** | 4 (10.81) | 0 (0.00) | 2 (0.20) | 1 (20.00) | 1 (9.09) | 0 (0.00) |
| **Nintedanib** | 4 (10.81) | 0 (0.00) | 2 (20.00) | 0 (0.00) | 1 (9.09) | 1 (20.00) |
| **Ramucirumab** | 3 (8.11) | 0 (0.00) | 3 (30.00) | 0 (0.00) | 0 (0.00) | 0 (0.00) |
| **Gemcitabine** | 3 (8.11) | 1 (16.67) | 1 (0.10) | 0 (0.00) | 0 (0.00) | 1 (20.00) |
| **Paclitaxel** | 2 (5.41) | 2 (33.33) | 0 (0.00) | 0 (0.00) | 0 (0.00) | 0 (0.00) |
| **Cisplatin** | 1 (2.70) | 0 (0.00) | 0 (0.00) | 1 (20.00) | 0 (0.00) | 0 (0.00) |
| **Afatinib** | 1 (2.70) | 0 (0.00) | 1 (0.10) | 0 (0.00) | 0 (0.00) | 0 (0.00) |
| **2L regimens, n (%)** | **37 (11.00)** | **6 (10.00)** | **10 (17.00)** | **5 (7%)** | **11 (16)** | **5 (8%)** |
| **Docetaxel** | 20 (54.05) | 3 (50.00) | 2 (20.00) | 3 (60.00) | 9 (81.82) | 3 (60.00) |
| **Docetaxel \| Nintedanib** | 4 (10.81) | 0 (0.00) | 2 (20.00) | 0 (0.00) | 1 (9.09) | 1 (20.00) |
| **Vinorelbine** | 3 (8.11) | 0 (0.00) | 1 (10.00) | 1 (20.00) | 1 (9.09) | 0 (0.00) |
| **Ramucirumab \| Docetaxel** | 3 (8.11) | 0 (0.00) | 3 (30.00) | 0 (0.00) | 0 (0.00) | 0 (0.00) |
| **Paclitaxel** | 2 (5.41) | 2 (33.33) | 0 (0.00) | 0 (0.00) | 0 (0.00) | 0 (0.00) |
| **Gemcitabine** | 2 (5.41) | 1 (16.67) | 0 (0.00) | 0 (0.00) | 0 (0.00) | 1 (20.00) |
| **Cisplatin** | 1 (2.70) | 0 (0.00) | 0 (0.00) | 1 (20.00) | 0 (0.00) | 0 (0.00) |
| **Gemcitabine \| Vinorelbine** | 1 (2.70) | 0 (0.00) | 1 (10.00) | 0 (0.00) | 0 (0.00) | 0 (0.00) |
| **Afatanib** | 1 (2.70) | 0 (0.00) | 1 (10.00) | 0 (0.00) | 0 (0.00) | 0 (0.00) |
| **3L treatments, n (%)** | **2 (1)** | **0 (0.00)** | **1 (2.00)** | **0 (0.00)** | **1 (1.00)** | **0 (0.00)** |
| **Vinorelbine** | 1 (50.00) | 0 (0.00) | 0 (0.00) | 0 (0.00) | 1 (1.00) | 0 (0.00) |
| **Nivolumab** | 1 (50.00) | 0 (0.00) | 1 (100.00) | 0 (0.00) | 0 (0.00) | 0 (0.00) |
| **3L regimens, n (%)** | **2 (1.00)** | **0 (0.00)** | **1 (2.00)** | **0 (0.00)** | **1 (1.00)** | **0 (0.00)** |
| **Vinorelbine** | 1 (50.00) | 0 (0.00) | 0 (0.00) | 0 (0.00) | 0 (0.00) | 0 (0.00) |
| **Nivolumab** | 1 (50.00) | 0 (0.00) | 1 (100.00) | 0 (0.00) | 1 (100.00) | 0 (0.00) |

*Includes Cisplatin | Gemcitabine | Pembrolizumab; Carboplatin | Pembrolizumab | Paclitaxel | Pemetrexed; Cisplatin | Pembrolizumab | Paclitaxel | Pemetrexed; Carboplatin | Gemcitabine | Pembrolizumab; Cisplatin | Pembrolizumab | Vinorelbine; Oxaliplatin | Pembrolizumab; Carboplatin | Docetaxel | Pembrolizumab; Cisplatin | Paclitaxel | Pembrolizumab | Pemetrexed

1L, first-line; 1LM, first-line maintenance; 2L, second-line; 3L, third-line; LOT, line of therapy

Supplementary **Table 4.** Treatment regimens per LOT by histology and PD-L1 TPS

|  | **Histology at diagnosis of advanced/metastatic NSCLC** | | | **PD-L1 status (by TPS) at index date** | | |
| --- | --- | --- | --- | --- | --- | --- |
|  | **Squamous  (N=97)** | **Non-squamous (N=219)** | **Mixed (N=6)** | **TPS negative (<1%)  (N=34)** | **TPS  1–49%  (N=108)** | **TPS ≥50%**  **(N=28)** |
| **1L induction regimen received, n (%)** | **97 (100.00)** | **219 (100.00)** | **6 (100.00)** | **34 (100.00)** | **108 (100.00)** | **28 (100.00)** |
| **Carboplatin \| Pembrolizumab \| Pemetrexed** | 6 (6.19) | 108 (49.32) | 3 (50.00) | 13 (38.24) | 33 (30.56) | 16 (57.14) |
| **Carboplatin \| Pembrolizumab \| Paclitaxel** | 65 (67.01) | 33 (15.07) | 0 (0.00) | 12 (35.29) | 37 (34.26) | 9 (32.14) |
| **Cisplatin \| Pembrolizumab \| Pemetrexed** | 1 (1.03) | 72 (32.88) | 1 (16.67) | 8 (23.53) | 30 (27.78) | 2 (7.14) |
| **Cisplatin \| Pembrolizumab** | 7 (7.22) | 2 (0.91) | 1 (16.67) | 0 (0.00) | 0 (0.00) | 0 (0.00) |
| **Cisplatin \| Pembrolizumab \| Paclitaxel** | 5 (5.15) | 2 (0.91) | 0 (0.00) | 1 (2.94) | 4 (3.70) | 0 (0.00) |
| **Carboplatin \| Pembrolizumab** | 5 (5.15) | 1 (0.46) | 0 (0.00) | 0 (0.00) | 1 (0.93) | 0 (0.00) |
| **Cisplatin \| Gemcitabine \| Pembrolizumab** | 2 (2.06) | 0 (0.00) | 1 (16.67) | 0 (0.00) | 2 (1.85) | 0 (0.00) |
| **Carboplatin \| Pembrolizumab \| Paclitaxel \| Pemetrexed** | 1 (1.03) | 0 (0.00) | 0 (0.00) | 0 (0.00) | 0 (0.00) | 0 (0.00) |
| **Cisplatin \| Pembrolizumab \| Paclitaxel \| Pemetrexed** | 1 (1.03) | 1 (0.46) | 0 (0.00) | 0 (0.00) | 0 (0.00) | 0 (0.00) |
| **Carboplatin \| Gemcitabine \| Pembrolizumab** | 1 (1.03) | 0 (0.00) | 0 (0.00) | 0 (0.00) | 0 (0.00) | 1 (3.57) |
| **Cisplatin \| Pembrolizumab \| Vinorelbine** | 1 (1.03) | 0 (0.00) | 0 (0.00) | 0 (0.00) | 0 (0.00) | 0 (0.00) |
| **Oxaliplatin \| Pembrolizumab** | 1 (1.03) | 0 (0.00) | 0 (0.00) | 0 (0.00) | 0 (0.00) | 0 (0.00) |
| **Carboplatin \| Docetaxel \| Pembrolizumab** | 1 (1.03) | 0 (0.00) | 0 (0.00) | 0 (0.00) | 1 (0.93) | 0 (0.00) |
| **Cisplatin \| Paclitaxel \| Pembrolizumab \| Pemetrexed** | 1 (1.03) | 0 (0.00) | 0 (0.00) | 0 (0.00) | 0 (0.00) | 0 (0.00) |
| **1LM regimens, n (%)** | **97 (100.00)** | **219 (100.00)** | **6 (100.00)** | **34 (100.00)** | **108 (100.00)** | **28 (100.00)** |
| **Pembrolizumab** | 90 (92.78) | 149 (68.04) | 5 (83.33) | 24 (70.59) | 84 (77.78) | 21 (75.00) |
| **Pembrolizumab \| Pemetrexed** | 4 (4.12) | 63 (28.77) | 1 (16.67) | 10 (20.41) | 22 (20.37) | 6 (21.43) |
| **Cisplatin \| Pembrolizumab \| Pemetrexed** | 0 (0.00) | 4 (1.83) | 0 (0.00) | 0 (0.00) | 0 (0.00) | 1 (3.57) |
| **Carboplatin \| Pembrolizumab \| Paclitaxel** | 1 (1.03) | 1 (0.46) | 0 (0.00) | 0 (0.00) | 1 (0.93) | 0 (0.00) |
| **Carboplatin \| Pembrolizumab \| Pemetrexed** | 0 (0.00) | 1 (0.46) | 0 (0.00) | 0 (0.00) | 0 (0.00) | 0 (0.00) |
| **Pembrolizumab \| Paclitaxel** | 1 (1.03) | 0 (0.00) | 0 (0.00) | 0 (0.00) | 1 (0.93) | 0 (0.00) |
| **Oxaliplatin \| Pembrolizumab** | 1 (1.03) | 0 (0.00) | 0 (0.00) | 0 (0.00) | 0 (0.00) | 0 (0.00) |
| **Carboplatin \| Pembrolizumab** | 0 (0.00) | 1 (0.46) | 0 (0.00) | 0 (0.00) | 0 (0.00) | 0 (0.00) |
| **2L regimens, n (%)** | **8 (8.00)** | **29 (13.00)** | **0 (0.00)** | **4 (12.00)** | **17 (16.00)** | **2 (7.00)** |
| **Docetaxel** | 2 (25.00) | 18 (62.07) | 0 (0.00) | 3 (75.00) | 10 (58.82) | 1 (50.00) |
| **Docetaxel \| Nintedanib** | 0 (0.00) | 4 (13.79) | 0 (0.00) | 0 (0.00) | 2 (11.76) | 0 (0.00) |
| **Vinorelbine** | 2 (25.00) | 1 (3.45) | 0 (0.00) | 1 (25.00) | 0 (0.00) | 0 (0.00) |
| **Ramucirumab \| Docetaxel** | 0 (0.00) | 3 (10.34) | 0 (0.00) | 0 (0.00) | 1 (5.88) | 0 (0.00) |
| **Paclitaxel** | 0 (0.00) | 2 (6.90) | 0 (0.00) | 0 (0.00) | 1 (5.88) | 1 (50.00) |
| **Gemcitabine** | 2 (25.00) | 0 (0.00) | 0 (0.00) | 0 (0.00) | 2 (11.76) | 0 (0.00) |
| **Cisplatin** | 0 (0.00) | 1 (3.45) | 0 (0.00) | 0 (0.00) | 0 (0.00) | 0 (0.00) |
| **Gemcitabine \| Vinorelbine** | 1 (12.50) | 0 (0.00) | 0 (0.00) | 0 (0.00) | 0 (0.00) | 0 (0.00) |
| **Afatanib** | 1 (12.50) | 0 (0.00) | 0 (0.00) | 0 (0.00) | 1 (5.88) | 0 (0.00) |
| **3L regimens, n (%)** | **0 (0.00)** | **2 (1.00)** | **0 (0.00)** | 0 (0.00) | **1 (1.00)** | 0 (0.00) |
| **Vinorelbine** | 0 (0.00) | 1 (50.00) | 0 (0.00) | 0 (0.00) | 1 (100.00) | 0 (0.00) |
| **Nivolumab** | 0 (0.00) | 1 (50.00) | 0 (0.00) | 0 (0.00) | 0 (0.00) | 0 (0.00) |

1L, first-line; 1LM, first-line maintenance; 2L, second-line; 3L, third-line; CI, confidence interval; LOT, line of therapy; NSCLC, non-small cell lung cancer; PD-L1, programmed death-ligand 1; TPS, tumor proportion score

Supplementary Table 5. DOT, TTD, and rwTTNT for each LOT, in the EU5 and by country

|  | **EU5  (N=322)** | **France (N=61)** | **Germany (N=60)** | **Italy  (N=73)** | **Spain  (N=67)** | **UK**  **(N=61)** |
| --- | --- | --- | --- | --- | --- | --- |
| **DOT*** |  |  |  |  |  |  |
| **1L induction, valid n** | 322 | 61 | 60 | 73 | 67 | 61 |
| **Median, months (CI)** | 3.0 (3.0, 3.0) | 3.9 (3.0, 4.0) | 3.0 (3.0, 3.9) | 3.0 (3.0, 3.9) | 3.0 (3.0, 3.9) | 3.0 (3.0, 3.0) |
| **1LM, valid n** | 322 | 61 | 60 | 73 | 67 | 61 |
| **Median, months (CI)** | 5.0 (3.9, 6.0) | 4.0 (2.9, 7,1) | 6.0 (6.0, 10.0) | 5.0 (2.0, 6.0) | 3.0 (1.9, 5.0) | 6.0 (3.0, 7.0) |
| **2L, valid n** | 37 | 6 | 10 | 5 | 11 | 5 |
| **Median, months (CI)** | 2.9 (1.9, 3.0) | 2.9 (0.5, NA) | 3.9 (0.5, 5.0) | 1.0 (0.9, NA) | 2.9 (1.9, 3.9) | 1.9 (1, NA) |
| **3L, valid n** | 2 | 0 | 1 | 0 | 1 | 0 |
| **Median, months (CI)** | 4.9 (1.9, NA) |  | 1.9 (NA, NA) |  | 8.0 (NA, NA) |  |
| **TTD^†^** |  |  |  |  |  |  |
| **1LM, valid n** | 312 | 61 | 58 | 72 | 60 | 61 |
| **Median, months (CI)** | 5.0 (4.0, 6.0) | 4.0 (2.9, 7.1) | 6.0 (5.0, 9.0) | 5.0 (2.0, 7.0) | 3.9 (2.0, 6.0) | 6.0 (3.0, 7.0) |
| **2L, valid n** | 37 | 6 | 10 | 5 | 11 | 5 |
| **Median, months (CI)** | 2.9 (1.9, 3.0) | 2.9 (0.5, NA) | 3.9 (0.5, 5.0) | 1.0 (0.9, NA) | 2.9 (1.9, 3.9) | 1.9 (1, NA) |
| **3L, valid n** | 2 | 0 | 1 | 0 | 1 | 0 |
| **Median, months (CI)** | 4.9 (1.9, NA) |  | 1.9 (NA, NA) |  | 8.0 (NA, NA) |  |
| **rwTTNT^‡^** |  |  |  |  |  |  |
| **1L induction to 1LM, valid n** | 322 | 61 | 60 | 73 | 67 | 61 |
| **Median, months (CI)** | 4.0 (3.9, 4.0) | 4.0 (3.9, 4.0) | 3.9 (3.0, 4.0) | 3.9 (3.9, 4.0) | 3.9 (3.0, 4.0) | 4.0 (3.9, 4.0) |
| **1LM to 2L, valid n** | 322 | 61 | 60 | 73 | 67 | 61 |
| **Median, months (CI)** | 14.0 (11, NA) | 13.1 (7.1, NA) | 15.1 (10, NA) | NA (10, NA) | 12.0 (9.1, NA) | 14.0 (8.1, NA) |
| **2L to 3L, valid n** | 37 | 6 | 10 | 5 | 11 | 5 |
| **Median, months (CI)** | 12.0 (5.0, NA) | NA (NA, NA) | NA (5, NA) | NA (NA, NA) | 12.0 (NA, NA) | NA (NA, NA) |

*Defined as the time (months) from LOT start date to LOT end date or censored event; †Defined as the time (months) from the start to end dates of a LOT that was indicated as discontinued in the case report form or censored event; ^‡^Defined as the time (months) from the first dose of a given LOT (start date of given LOT; inclusive) until the first dose of the next distinct LOT (start date of next consecutive LOT, exclusive) or censored event. 1L, first-line, 1LM, first-line maintenance; 2L, second line; 3L, third line; CI, confidence interval; DOT, duration of treatment; LOT, line of treatment; NA, not available; rwTTNT, real-world time to next treatment; TTD, time to discontinuation.

Supplementary Table 6. DOT, TTD, and rwTTNT for each LOT by response to 1L induction and by CNS/brain metastases status (at index date)

|  | **Response to 1L induction** | | **Presence of CNS/brain metastases at index date** | | |
| --- | --- | --- | --- | --- | --- |
|  | **PR or CR**  **(N=235)** | **SD**  **(N=87)** | **Yes**  **(N=18)** | **No**  **(N=288)** | **Unknown**  **(N=16)** |
| **DOT*** |  |  |  |  |  |
| **1L induction, valid n** | **235** | **87** | **18** | **288** | **16** |
| **Median, months (CI)** | 3.0 (3.0, 3.9) | 3.0 (3.0, 3.0) | 3.0 (3.0, 4.0) | 3.0 (3.0, 3.0) | 3.0 (3.9, 4.0) |
| **1LM, valid n** | **235** | **87** | **18** | **288** | **16** |
| **Median, months (CI)** | 6.0 (5.0, 7.0) | 2.0 (1.0, 3.9) | 2.0 (1.0, 4.0) | 6.0 (5.0, 6.0) | 2.9 (1.0, 3.0) |
| **2L, valid n** | **27** | **10** | **0** | **36** | **1** |
| **Median, months (CI)** | 2.0 (1.9, 3.0) | 2.9 (0.5, 3.0) | 0 | 2.9 (1.9, 3.0) | 1.0 (NA, NA) |
| **3L, valid n** | **2** | **0** | **0** | **2** | **0** |
| **Median, months (CI)** | 4.9 (1.9, NA) | 0 | 0 | 4.9 (1.9, NA) | 0 |
| **TTD^†^** |  |  |  |  |  |
| **1LM, valid n** | **225** | **87** | **18** | **278** | **16** |
| **Median, months (CI)** | 6.0 (5.0, 7.1) | 2.0 (1.0, 3.9) | 2.0 (1.0, 4.0) | 6.0 (5.0, 7.0) | 2.0 (1.0, 3.0) |
| **2L, valid n** | **27** | **10** | **0** | **36** | **1** |
| **Median, months (CI)** | 2.0 (1.9, 3.0) | 2.9 (0.5, 3.0) | 0 | 2.9 (1.9, 3.0) | 1.0 (NA, NA) |
| **3L, valid n** | **2** | **0** | **0** | **2** | **0** |
| **Median, months (CI)** | 4.9 (1.9, NA) | 0 | 0 | 4.9 (1.9, NA) | 0 |
| **rwTTNT^‡^** |  |  |  |  |  |
| **1L induction to 1LM, valid n** | **235** | **87** | **18** | **288** | **16** |
| **Median, months (CI)** | 4.0 (3.9, 4.0) | 3.9 (3.0, 4.0) | 4.0 (3.0, 5.0) | 3.9 (3.0, 4.0) | 4.0 (2.9, 4.9) |
| **1LM to 2L, valid n** | **235** | **87** | **18** | **288** | **16** |
| **Median, months (CI)** | 13.1 (11.0, 36.0) | NA (7.1, NA) | NA (NA, NA) | 14.0 (11.0, NA) | NA (5.0, NA) |
| **2L to 3L, valid n** | **27** | **10** | **0** | **36** | **1** |
| **Median, months (CI)** | 12.0 (5.0, NA) | NA (NA, NA) | 0 | 12.0 (5.0, NA) | NA (NA, NA) |

*Defined as the time (months) from LOT start date to LOT end date or censored event; †Defined as the time (months) from the start to end dates of a LOT that was indicated as discontinued in the case report form or censored event; ^‡^Defined as the time (months) from the first dose of a given LOT (start date of given LOT; inclusive) until the first dose of the next distinct LOT (start date of next consecutive LOT, exclusive) or censored event. 1L, first-line, 1LM, first-line maintenance; 2L, second line; 3L, third line; CI, confidence interval; CNS, central nervous system; CR, complete response; DOT, duration of treatment; NA, not available, PR, partial response; rwTTNT, real-world time to next treatment; SD, stable disease; TTD, time to discontinuation.

Supplementary Table 7. DOT, TTD, and rwTTNT for each LOT by histology and PD-L1 TPS

|  | **Histology at diagnosis of advanced/metastatic NSCLC** | | | **PD-L1 status (by TPS) at index date** | | |
| --- | --- | --- | --- | --- | --- | --- |
|  | **Squamous (N=97)** | **Non-squamous (N=219)** | **Mixed (N=6)** | **TPS negative (<1%)  (N=34)** | **TPS  ≥1–49%  (N=108)** | **TPS ≥50%**  **(N=28)** |
| **DOT*** |  |  |  |  |  |  |
| **1L induction, valid n** | 97 | 219 | 6 | 34 | 108 | 28 |
| **Median, months (CI)** | 3.0 (3.0, 3.0) | 3.0 (3.0, 3.9) | 3.5 (2.0, NA) | 3.0 (2.0, 3.9) | 3.0 (3.0, 3.0) | 3.0 (3.0, 4.9) |
| **1LM, valid n** | 97 | 219 | 6 | 34 | 108 | 28 |
| **Median, months (CI)** | 4.0 (2.0, 6.0) | 6.0 (4.0, 7.0) | 5.0 (0.5, NA) | 3.0 (1.9, 7.0) | 6.0 (3.9, 7.0) | 6.0 (2.0, 7.0) |
| **2L, valid n** | 8 | 29 |  | 4 | 17 | 2 |
| **Median, months (CI)** | 1.9 (1, NA) | 2.9 (1.9, 3) | 0 | 2.0 (0.9, NA) | 3.0 (1.0, 3.9) | 2.9 (NA, NA) |
| **3L, valid n** | 0 | 2 | 0 | 0 | 1 | 0 |
| **Median, months (CI)** |  | 4.9 (1.9, NA) |  |  | 8.0 (NA, NA) |  |
| **TTD^†^** |  |  |  |  |  |  |
| **1LM, valid n** | 96 | 210 | 6 | 32 | 106 | 27 |
| **Median, months (CI)** | 4.0 (2.0, 6.0) | 6.0 (4.0, 7.0) | 5.0 (0.5, NA) | 3.0 (1.9, 6.0) | 6.0 (3.9, 7.0) | 4.0 (2.0, 7.0) |
| **2L, valid n** | 8 | 29 | 0 | 4 | 17 | 2 |
| **Median, months (CI)** | 1.9 (1.0, NA) | 2.9 (1.9, 3.0) |  | 2.0 (0.9, NA) | 3.0 (1.0, 3.9) | 2.9 (NA, NA) |
| **3L, valid n** | 0 | 2 | 0 | 0 | 1 | 0 |
| **Median, months (CI)** |  | 4.9 (1.9, NA) |  |  | 8.0 (NA, NA) |  |
| **rwTTNT^‡^** |  |  |  |  |  |  |
| **1L induction to 1LM, valid n** | 97 | 219 | 6 | 28 | 108 | 34 |
| **Median, months (CI)** | 3.9 (3.0, 4.0) | 4.0 (3.9, 4.0) | 4.5 (2, NA) | 4.0 (3.0, 4.0) | 3.9 (3.0, 4.0) | 4.0 (3.0, 4.9) |
| **1LM to 2L, valid n** | 97 | 219 | 6 | 28 | 108 | 34 |
| **Median, months (CI)** | 14.0 (9.1, NA) | 13.1 (11.0, NA) | NA (NA, NA) | NA (8.1, NA) | 12.0 (11, NA) | NA (7.1, NA) |
| **2L to 3L, valid n** | 8 | 29 | 0 | 17 | 4 | 0 |
| **Median, months (CI)** | NA (NA, NA) | 12.0 (5.0, NA) |  | NA (NA, NA) | 12 (NA, NA) |  |

*Defined as the time (months) from LOT start date to LOT end date or censored event; †Defined as the time (months) from the start to end dates of a LOT that was indicated as discontinued in the case report form or censored event; ^‡^Defined as the time (months) from the first dose of a given LOT (start date of given LOT; inclusive) until the first dose of the next distinct LOT (start date of next consecutive LOT, exclusive) or censored event. 1L, first-line; 1LM, first-line maintenance; 2L, second line; 3L, third line; CI, confidence interval; DOT, duration of treatment; LOT, line of therapy; NA, not available, NSCLC, non-small cell lung cancer; PD-L1, programmed death-ligand 1; rwTTNT, real-world time to next treatment; TPS, tumor proportion score; TTD, time to discontinuation

Supplementary Table 8. DOT, TTD, and rwTTNT for each LOT in patients with non-squamous histology, by use of pemetrexed in 1LM regimen

|  | **Pemetrexed included in 1LM regimen** | |
| --- | --- | --- |
|  | **Yes**  **(N=68)** | **No**  **(N=151)** |
| **DOT*** |  |  |
| **1L induction, valid n** | 68 | 151 |
| **Median, months (CI)** | 3.0 (3.0, 3.0) | 3.0 (3.0, 3.9) |
| **1LM, valid n** | 63 | 147 |
| **Median, months (CI)** | 6.0 (3.0, 7.0) | 6.0 (4.0, 7.0) |
| **2L, valid n** | 14 | 15 |
| **Median, months (CI)** | 2.9 (0.9, 3.0) | 2.9 (1.0, 3.9) |
| **3L, valid n** | 1 | 1 |
| **Median, months (CI)** | 8.0 (NA, NA) | 1.9 (NA, NA) |
| **TTD^†^** |  |  |
| **1LM, valid n** | 63 | 147 |
| **Median, months (CI)** | 6.0 (3.0, 7.0) | 6.0 (4.0, 7.0) |
| **2L, valid n** | 14 | 15 |
| **Median, months (CI)** | 2.9 (0.9, 3.0) | 2.9 (1.0, 3.9) |
| **3L, valid n** | 1 | 1 |
| **Median, months (CI)** | 8.0 (NA, NA) | 1.9 (NA, NA) |

*Defined as the time (months) from LOT start date to LOT end date or censored event; †Defined as the time (months) from the start to end dates of a LOT that was indicated as discontinued in the case report form or censored event; 1L, first-line; 1LM, first-line maintenance; 2L, second line; 3L, third line; CI, confidence interval; DOT, duration of treatment; LOT, line of therapy; NA, not available; rwTTNT, real-world time to next treatment; TTD, time to discontinuation.

Supplementary Table 9. Reasons for LOT discontinuation in EU5 and by country

|  | **EU5  (N=322)** | **France (N=61)** | **Germany (N=60)** | **Italy  (N=73)** | **Spain  (N=67)** | **UK**  **(N=61)** |
| --- | --- | --- | --- | --- | --- | --- |
| **Discontinued 1LM therapy, n/N (%)** | **185/322 (57.45)** | **31/61  (50.82)** | **33/60  (55.00)** | **45/73  (61.64)** | **46/67  (68.66)** | **30/61  (49.18)** |
| **Of whom, reasons for discontinuation were:** | | | | | | |
| **Course complete, n (%)** | 10 (5.41) | 0 (0.00) | 2 (6.10) | 1 (2.22) | 7 (15.22) | 0 (0.00) |
| **Poor ECOG PS, n (%)** | 17 (9.19) | 6 (19.35) | 3 (9.10) | 2 (4.44) | 3 (6.52) | 3 (10.00) |
| **Adverse event(s), n (%)** | 5 (2.70) | 0 (0.00) | 1 (3.00) | 0 (0.00) | 3 (6.52) | 1 (3.33) |
| **Distant progression/relapse: CNS/brain progression only, n (%)** | 16 (8.65) | 2 (6.45) | 2 (6.10) | 3 (6.67) | 5 (10.87) | 4 (13.33) |
| **Distant progression/relapse: CNS/brain and extracranial progression, n (%)** | 44 (23.78) | 7 (22.58) | 5 (15.20) | 14 (31.11) | 10 (21.74) | 8 (26.67) |
| **Local progression/relapse, n (%)** | 42 (22.70) | 11 (35.48) | 10 (30.30) | 6 (13.33) | 7 (15.22) | 8 (26.67) |
| **Withdrawal of consent/patient’s choice, n (%)** | 11 (5.95) | 0 (0.00) | 3 (9.10) | 7 (15.56) | 0 (0.00) | 1 (3.33) |
| **Risk to the patient, n (%)** | 6 (3.24) | 2 (6.45) | 1 (3.00) | 1 (2.22) | 1 (2.17) | 1 (3.33) |
| **Severe non-compliance with treatment, n (%)** | 1 (0.54) | 0 (0.00) | 1 (3.00) | 0 (0.00) | 0 (0.00) | 0 (0.00) |
| **Lost to follow-up, n (%)** | 3 (1.62) | 1 (3.23) | 0 (0.00) | 0 (0.00) | 2 (4.35) | 0 (0.00) |
| **Side effects, n (%)** | 4 (2.16) | 0 (0.00) | 0 (0.00) | 0 (0.00) | 3 (6.52) | 1 (3.33) |
| **Death, n (%)** | 68 (36.76) | 11 (35.48) | 14 (42.40) | 17 (37.78) | 14 (30.43) | 12 (40.00) |
| **Other, n (%)** | 1 (0.54) | 0 (0.00) | 0 (0.00) | 0 (0.00) | 1 (2.17) | 0 (0.00) |
| **Discontinued 2L therapy, n/N (%)** | **31/37 (83.78)** | **6/6 (100.00)** | **8/10 (80.00)** | **3/5 (60.00)** | **9/11 (81.81)** | **5/5 (100.00)** |
| **Of whom, reasons for discontinuation were:** | | | | | | |
| **Course complete** | 0 (0.00) | 0 (0.00) | 0 (0.00) | 0 (0.00) | 0 (0.00) | 0 (0.00) |
| **Poor ECOG PS** | 9 (29.03) | 3 (50.00) | 1 (12.50) | 1 (33.33) | 2 (22.22) | 2 (40.00) |
| **Adverse event(s)** | 0 (0.00) | 0 (0.00) | 0 (0.00) | 0 (0.00) | 0 (0.00) | 0 (0.00) |
| **Distant progression/relapse: CNS/brain progression only** | 4 (12.90) | 1 (16.67) | 1 (12.50) | 0 (0.00) | 1 (11.11) | 1 (20.00) |
| **Distant progression/relapse: CNS/brain and extracranial progression** | 11 (35.48) | 3 (50.00) | 2 (25.00) | 1 (33.33) | 4 (44.44) | 1 (20.00) |
| **Local progression/relapse** | 10 (32.26) | 1 (16.67) | 5 (62.50) | 0 (0.00) | 3 (33.33) | 1 (20.00) |
| **Withdrawal of consent/patient’s choice** | 1 (3.23) | 0 (0.00) | 0 (0.00) | 0 (0.00) | 0 (0.00) | 1 (20.00) |
| **Risk to the patient** | 0 (0.00) | 0 (0.00) | 0 (0.00) | 0 (0.00) | 0 (0.00) | 0 (0.00) |
| **Severe non-compliance with treatment** | 0 (0.00) | 0 (0.00) | 0 (0.00) | 0 (0.00) | 0 (0.00) | 0 (0.00) |
| **Lost to follow-up** | 0 (0.00) | 0 (0.00) | 0 (0.00) | 0 (0.00) | 0 (0.00) | 0 (0.00) |
| **Side effects** | 2 (6.45) | 0 (0.00) | 1 (12.50) | 0 (0.00) | 1 (11.11) | 0 (0.00) |
| **Death** | 6 (19.35) | 2 (33.33) | 1 (12.50) | 1 (33.33) | 1 (11.11) | 1 (20.00) |
| **Other** | 0 (0.00) | 0 (0.00) | 0 (0.00) | 0 (0.00) | 0 (0.00) | 0 (0.00) |

1LM, first-line maintenance; 2L, second-line; CNS, central nervous system; ECOG PS, Eastern Oncology Cooperative Group performance status; LOT, line of therapy

Supplementary Table 10. Reasons for LOT discontinuation by histology and PD-L1 TPS

|  | **Histology at diagnosis of advanced/metastatic NSCLC** | | | **PD-L1 status (by TPS) at index date** | | |
| --- | --- | --- | --- | --- | --- | --- |
|  | **Squamous  (N=97)** | **Non-squamous (N=219)** | **Mixed (N=6)** | **TPS negative (<1%)  (N=34)** | **TPS  ≥1–49%  (N=108)** | **TPS ≥50%**  **(N=28)** |
| **Discontinued 1LM therapy, n/N (%)** | **59/97 (60.82)** | **122/219 (55.71)** | **4/6 (66.67)** | **27/34 (79.41)** | **56/108 (51.85)** | **13/28 (46.43)** |
| **Of whom, reasons for discontinuation were:** | | | | | | |
| **Course complete** | 1 (1.69) | 9 (7.38) | 0 (0.00) | 2 (7.41) | 2 (3.57) | 1 (7.69) |
| **Poor ECOG PS** | 7 (11.86) | 10 (8.20) | 0 (0.00) | 3 (11.11) | 4 (7.14) | 1 (7.69) |
| **Adverse event(s)** | 2 (3.39) | 3 (2.46) | 0 (0.00) | 0 (0.00) | 2 (3.57) | 0 (0.00) |
| **Distant progression/relapse: CNS/brain progression only** | 7 (11.86) | 9 (7.38) | 0 (0.00) | 3 (11.11) | 5 (8.93) | 1 (7.69) |
| **Distant progression/relapse: CNS/brain and extracranial progression** | 12 (20.34) | 31 (25.41) | 1 (25.00) | 10 (37.04) | 13 (23.21) | 4 (30.77) |
| **Local progression/relapse** | 19 (32.20) | 23 (18.85) | 0 (0.00) | 4 (14.81) | 16 (28.57) | 1 (7.69) |
| **Withdrawal of consent/patient’s choice** | 3 (5.08) | 8 (6.56) | 0 (0.00) | 2 (7.41) | 4 (7.14) | 1 (7.69) |
| **Risk to the patient** | 1 (1.69) | 5 (4.10) | 0 (0.00) | 0 (0.00) | 1 (1.79) | 1 (7.69) |
| **Severe non-compliance with treatment** | 0 (0.00) | 1 (0.82) | 0 (0.00) | 0 (0.00) | 1 (1.79) | 0 (0.00) |
| **Lost to follow-up** | 0 (0.00) | 3 (2.46) | 0 (0.00) | 0 (0.00) | 0 (0.00) | 0 (0.00) |
| **Side effects** | 0 (0.00) | 4 (3.28) | 0 (0.00) | 0 (0.00) | 1 (1.79) | 0 (0.00) |
| **Death** | 26 (44.07) | 40 (32.79) | 2 (50.00) | 9 (33.33) | 21 (37.50) | 4 (30.77) |
| **Other** | 0 (0.00) | 0 (0.00) | 1 (25.00) | 0 (0.00) | 0 (0.00) | 1 (7.69) |
| **Discontinued 2L therapy, n (%)** | **6/8 (75.00)** | **25/29 (86.21)** | **0/0 (0.00)** | **3/4 (75.00)** | **14/17 (82.35)** | **2/2 (100.00)** |
| **Of whom, reasons for discontinuation were:** | | | | | | |
| **Course complete** | 0 (0.00) | 0 (0.00) | 0 (0.00) | (0.00) | (0.00) | (0.00) |
| **Poor ECOG PS** | 2 (33.33) | 7 (28.00) | 0 (0.00) | 1 (33.33) | 3 (21.43) | 2 (100.00) |
| **Adverse event(s)** | 0 (0.00) | 0 (0.00) | 0 (0.00) | (0.00) | (0.00) | (0.00) |
| **Distant progression/relapse: CNS/brain progression only** | 0 (0.00) | 4 (16.00) | 0 (0.00) | 1 (33.33) | (0.00) | (0.00) |
| **Distant progression/relapse: CNS/brain and extracranial progression** | 1 (16.67) | 10 (40.00) | 0 (0.00) | 1 (33.33) | 4 (28.57) | 2 (100.00) |
| **Local progression/relapse** | 2 (33.33) | 8 (32.00) | 0 (0.00) | (0.00) | 5 (35.71) | 1 (50.00) |
| **Withdrawal of consent/patient’s choice** | 1 (16.67) | 0 (0.00) | 0 (0.00) | (0.00) | 1 (7.14) | (0.00) |
| **Risk to the patient** | 0 (0.00) | 0 (0.00) | 0 (0.00) | (0.00) | (0.00) | (0.00) |
| **Severe non-compliance with treatment** | 0 (0.00) | 0 (0.00) | 0 (0.00) | (0.00) | (0.00) | (0.00) |
| **Lost to follow-up** | 0 (0.00) | 0 (0.00) | 0 (0.00) | (0.00) | (0.00) | (0.00) |
| **Side effects** | 0 (0.00) | 2 (8.00) | 0 (0.00) | (0.00) | 2 (14.29) | (0.00) |
| **Death** | 3 (50.00) | 3 (12.00) | 0 (0.00) | (0.00) | 4 (28.57) | (0.00) |
| **Other** | 0 (0.00) | 0 (0.00) | 0 (0.00) | (0.00) | (0.00) | (0.00) |

1LM, first-line maintenance; 2L, second-line; CNS, central nervous system; ECOG PS, Eastern Oncology Cooperative Group performance status; NSCLC, non-small cell lung cancer; PD-L1, programmed death-ligand 1; TPS, tumor proportion score

# Supplementary Figures

Supplementary Figure 1. Timing of study measures for eligible patients

1L, first-line; CNS, central nervous system; CR, complete response; DoR, duration of response; ECOG, Eastern Cooperative Oncology Group; LOT, line of therapy; NSCLC, non-small cell lung cancer; ORR, objective response rate; PD-L1, programmed death ligand-1; PR, partial response.

**Supplementary Figure 2.** Treatment regimens used in (A) 1LM by histology, (B) 2L therapy by histology, (C) 1LM by PD-L1 TPS and (D) 2L therapy by PD-L1 TPS

1LM, first-line maintenance; 2L, second-line; NSQ, non-squamous; PD-L1, programmed death-ligand 1; SQ, squamous; TPS, tumor proportion score
